# Prophylactic NAC promoted hematopoietic reconstitution by improving endothelial cells after haploidentical HSCT: a phase 3, open-label randomized trial

**DOI:** 10.1186/s12916-022-02338-9

**Published:** 2022-04-27

**Authors:** Yu Wang, Yuan Kong, Hong-Yan Zhao, Yuan-Yuan Zhang, Ya-Zhe Wang, Lan-Ping Xu, Xiao-Hui Zhang, Kai-Yan Liu, Xiao-Jun Huang

**Affiliations:** 1 Peking University People’s Hospital, Peking University Institute of Hematology, Beijing Key Laboratory of Hematopoietic Stem Cell Transplantation, Collaborative Innovation Center of Hematology, Peking University, Beijing, China; 2 Peking-Tsinghua Center for Life Sciences, Academy for Advanced Interdisciplinary Studies, Peking University, Beijing, China

**Keywords:** *N*-Acetyl-L-cysteine, Poor hematopoietic reconstitution, Allogeneic hematopoietic stem cell transplantation, Endothelial cells, Bone marrow microenvironment

## Abstract

**Background:**

Poor graft function (PGF) or prolonged isolated thrombocytopenia (PT), which are characterized by pancytopenia or thrombocytopenia, have become serious complications after allogeneic hematopoietic stem cell transplantation (allo-HSCT). Our previous single-arm trial suggests that *N*-acetyl-L-cysteine (NAC) prophylaxis reduced PGF or PT after allo-HSCT. Therefore, an open-label, randomized, phase 3 trial was performed to investigate the efficacy and tolerability of NAC prophylaxis to reduce PGF or PT after allo-HSCT.

**Methods:**

A phase 3, open-label randomized trial was performed. Based on the percentage of CD34^+^VEGFR2 (CD309)^+^ endothelial cells (ECs) in bone marrow (BM) detected by flow cytometry at 14 days before conditioning, patients aged 15 to 60 years with acute leukemia undergoing haploidentical HSCT were categorized as low-risk (EC ≥ 0.1%) or high-risk (EC < 0.1%); patients at high risk were randomly assigned (2:1) to receive NAC prophylaxis or nonprophylaxis. The primary endpoint was PGF and PT incidence at +60 days post-HSCT.

**Results:**

Between April 18, 2019, and June 24, 2021, 120 patients with BM EC <0.1% were randomly assigned for NAC (group A, *N* = 80) or nonprophylaxis (group B, *N* = 40), and 105 patients with EC≥0.1% (group C) were also analyzed. The +60 days incidence of PGF and PT was 7.5% (95% CI, 1.7 to 13.3%) and 22.5% (95% CI, 9.1 to 35.9%) in group A and group B (hazard ratio, 0.317; 95% CI, 0.113 to 0.890; *P =* 0.021) and 11.4% (95% CI, 5.2 to 17.6%) in group C (hazard ratio, 0.643; 95% CI, 0.242 to 1.715; *P =* 0.373). Consistently, NAC prophylaxis gradually improved BM ECs and CD34^+^ cells in group A, whereas reduced their reactive oxygen species (ROS) levels post-HSCT. Within 60 days post-HSCT, the most common grade 3 to 5 adverse events for the NAC and control groups were infections (19/80 [24%] vs. 10/40 [25%]) and gastrointestinal adverse events (16/80 [20%] vs. 7/40 [18%]). There were no treatment-related deaths.

**Conclusions:**

*N*-Acetyl-L-cysteine prophylaxis can prevent the occurrence of poor hematopoietic function and is well tolerated in haploidentical HSCT. It may offer a potential pathogenesis-oriented therapeutic approach for patients with poor hematopoietic function.

**Trial registration:**

This trial was registered at ClinicalTrials.gov as #NCT03967665.

**Supplementary Information:**

The online version contains supplementary material available at 10.1186/s12916-022-02338-9.

## Background

Rapid and stable hematopoiesis recovery is a prerequisite for systemic and successful therapy in cancer patients [1, 2]. Moreover, poor hematopoietic function is a common feature of patients with bone marrow (BM) failure diseases, such as poor graft function (PGF) or prolonged isolated thrombocytopenia (PT) after allogeneic hematopoietic stem cell transplantation (allo-HSCT) [3–9], aplastic anemia [10, 11], and myelodysplastic syndromes [12–14]. Consequently, it is imperative to investigate how to promote hematopoiesis recovery in patients with poor hematopoietic function.

The specialized BM microenvironment is critical for the regulation of hematopoietic stem cells (HSCs) [15–19]. As a crucial element of the BM microenvironment, accumulating evidence indicates that endothelial cells (ECs) play essential roles in regulating hematopoiesis [20–25]. With the rapid development of haploidentical-HSCT (haplo-HSCT), poor hematopoietic function including PGF and PT [3–9], which is characterized by pancytopenia or thrombocytopenia, has become a serious threat after allo-HSCT because of the increased risk of infections and bleeding, hospitalization, even with worse health-related quality-of-life. Recently, our serial studies demonstrated that reduced and dysfunctional BM ECs, which are characterized by increased reactive oxygen species (ROS), induce the exhaustion of successfully engrafted donor HSCs, ultimately leading to the occurrence of poor hematopoietic function after allo-HSCT [3–7]. Therefore, BM EC dysfunction is the underlying pathogenesis in patients with poor hematopoietic function [3–7]. As an ROS scavenger, *N*-acety-L-cysteine (NAC) is widely used as an antioxidant and a mucolytic drug without significant side effects. NAC could enhance defective hematopoiesis by repairing the dysfunctional BM ECs of patients with PGF or PT in vitro [4, 7].

Considerable studies reported that the level of CD34^+^CD309^+^ EC detected by flow cytometry is a well-established prediction marker to identify patients at increased cardiovascular risk [26, 27]. Our recent single-arm trial [5] identified that BM CD34^+^CD309^+^ EC < 0.1% pre-haplo-HSCT is an independent risk factor for the occurrence of PGF or PT. Moreover, prophylactic oral NAC reduced the incidence of PGF or PT in EC < 0.1% group by improving BM ECs [5].

Therefore, we designed a phase 3, open-label randomized trial to validate the single-arm findings and to support the logical theory of novel BM microenvironment-directed therapies to promote hematopoietic reconstitution in patients with poor hematopoietic function.

## Methods

### Study design and participants

**Fig. 1:**
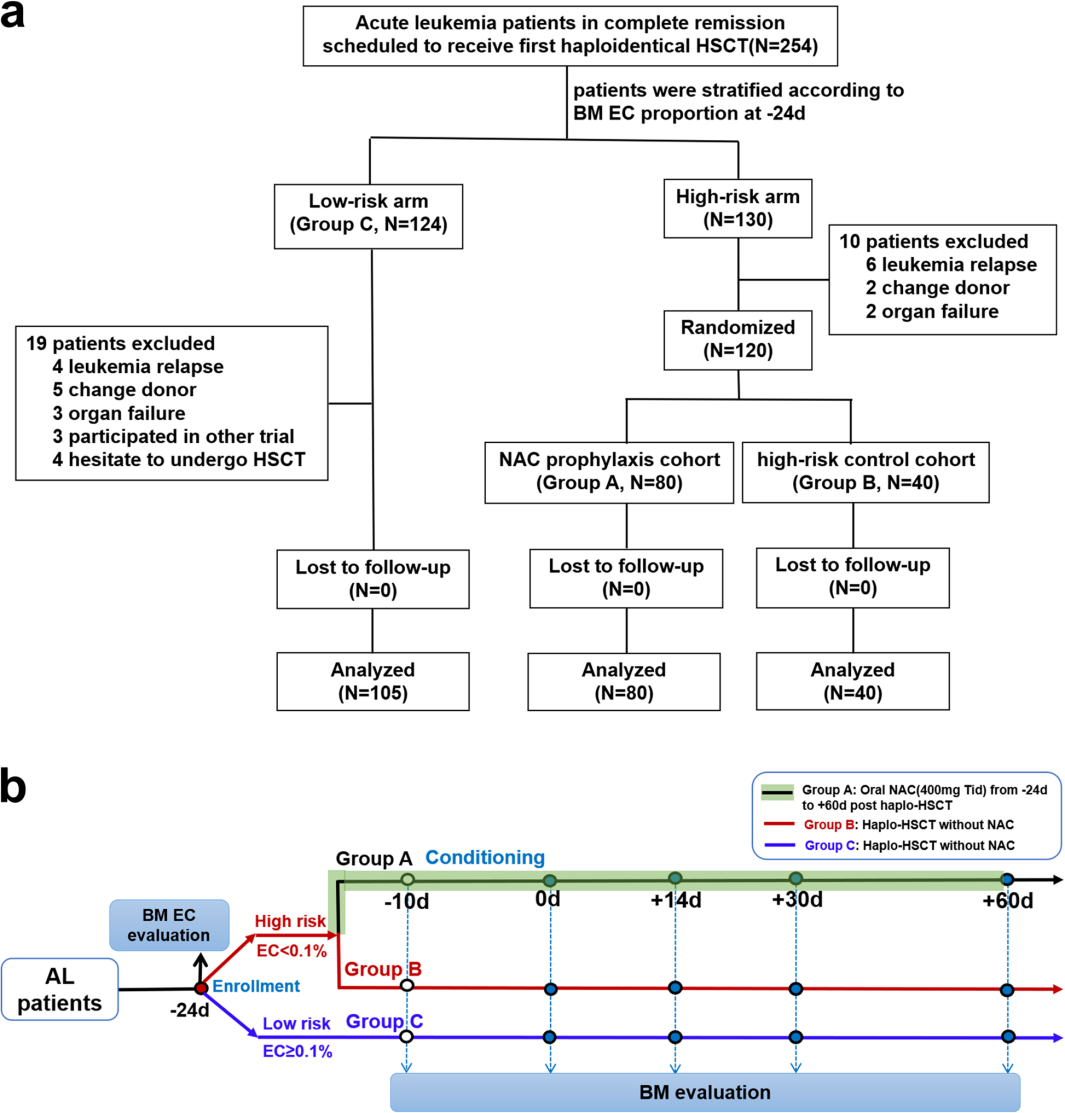
Trial flow chart

This phase 3, open-label randomized trial was performed at Peking University People’s Hospital between April 2019 and June 2021. Consecutive patients with acute leukemia (AL) undergoing first HSCT from haploidentical donors were eligible if they met the following criteria: (1) 15 to 60 years old with Eastern Cooperative Oncology Group (ECOG) performance status 0 to 2, (2) complete remission (CR) before HSCT, and (3) based on the percentage of BM ECs detected by flow cytometry at 14 days before conditioning (−24 days; detected by the same BM sample for morphology examination), patients were categorized as low-risk (EC ≥ 0.1%) or high-risk (EC < 0.1%); high-risk patients were randomly assigned (Fig. 1a). The AL diagnosis and CR identification was according to WHO criteria. Patients were excluded from the study if they had hypersensitivity to NAC, bronchial asthma, ejection fraction < 50%, creatinine ≥ 1.5 times the upper limit of normal (ULN), and total bilirubin or aminotransferase ≥ 2 times the ULN (detailed information on eligibility criteria is available in the study protocol, see Additional file 2).

As shown in Fig. 1a, a total of 120 high-risk (EC < 0.1%) patients were randomly assigned to receive NAC prophylaxis (group A, *N* = 80) or not to receive NAC prophylaxis (group B, *N* = 40), and low-risk (EC ≥ 0.1%) (group C, *N* = 105) patients were included in the final analysis.

The study protocol was approved by the ethics committee review board of Peking University People’s Hospital, and written informed consent was obtained from the subjects in accordance with the Declaration of Helsinki. This trial was registered at www.clinicaltrials.gov as # NCT03967665.

### Randomization and masking

As shown in Fig. 1b, patients with AL in CR scheduled to undergo first HSCT from haploidentical donor were screened for eligibility, which was performed ≤ 3 days prior to randomization (at 14 days before conditioning (−24 days)). Enrolled subjects were stratified as high-risk (EC < 0.1%) or low-risk (EC ≥ 0.1%, group C) for developing PGF or PT according to the previously identified percentage of BM ECs pre-haplo-HSCT [5]. High-risk patients were randomly allocated in a 2:1 ratio to receive NAC prophylaxis (group A) or nonprophylaxis (group B) at 14 days before conditioning (−24 days). Randomization was performed with permuted blocks (block size four) and conducted by an interactive web-based response system (IWRS). The computer-generated randomization codes were sent to the IWRS vendor to implement the randomization. Study site staff recruited participants. The next assignment in the sequence was concealed. The investigators or subjects were not masked to assignment. The outcome assessments and data analysis were undertaken in a masked pattern.

### Procedures

As previously described [4–7, 26–28], BM ECs were identified by mouse anti-human CD34, CD45, and vascular endothelial growth factor receptor 2 (VEGFR2, CD309) monoclonal antibodies (Becton Dickinson Biosciences, San Jose, CA) and analyzed using a BD LSRFortessa cell analyzer (Becton Dickinson). BM ECs were quantified by the percentages of CD34^+^CD309^+^ cells in total BM mononuclear cells (BMMNCs). The functions of BM ECs, including intracellular ROS levels, double-positive staining with both Dil-acetylated low-density lipoprotein (DiI-AcLDL) and fluorescein isothiocyanate–labeled Ulex Europaeus Agglutinin-1 (FITC-UEA-1), tube formation and migration assays, were analyzed pre- and post-HSCT as previously reported [4–7, 28].

Donor selection and human leukocyte antigen typing were performed as previously described [29]. All patients were given myeloablative chemotherapy-based conditioning regimen for a total of 10 days which included cytarabine (Ara-C, 4 g/m^2^/day, intravenously (i.v.), days −10 and −9), busulfan (Bu, 3.2 mg/kg/day, i.v., days −8 to −6), cyclophosphamide (Cy, 1.8 g/m^2^/day, i.v., days −5 and −4), and simustine (250 mg/m^2^, orally, day −3), plus antithymocyte globulin (ATG, 2.5 mg/kg/day, i.v., days −5 to −2), and received cyclosporin A (CsA), methotrexate (MTX), and mycophenolate (MMF) for graft-versus-host disease (GVHD) prophylaxis [30].

After enrollment, patients in group A were scheduled for NAC prophylaxis from 14 days before conditioning (−24 days) until +60 days post-HSCT. The initial dose of NAC was 400 mg orally three times daily (TID). For group B patients, NAC were not administered before +60 days post-HSCT. For patients in all the three groups, neither thrombopoietin (TPO) nor TPO receptor agonists (TPO-RAs) were administered before +60 days post-HSCT.

### Outcomes

The primary endpoint was the incidence of PGF or PT. Secondary endpoints included cumulative incidences of leukemia relapse (CIR), GVHD, nonrelapse mortality (NRM), leukemia-free survival (LFS), overall survival (OS), and adverse events (AEs).

PGF [3, 5–8] was defined as the presence of 2 or 3 cytopenic counts (absolute neutrophil count (ANC) ≤ 0.5×10^9^/L, platelet ≤ 20×10^9^/L, or hemoglobin ≤ 70g/L) for at least 3 consecutive days post-HSCT with a transfusion requirement related to hypoplastic-aplastic BM in the presence of complete donor chimerism (CDC) without disease relapse. Primary PGF was identified as the failure to achieve initial reconstitution by +28d post-HSCT. Secondary PGF was defined as the fulfillment of the criteria of PGF after reconstitution [31]. PT [4, 9, 32, 33] was defined as a platelet count less than 20×10^9^/L or a dependence on platelet transfusion with the engraftment of other cell lines (ANC > 0.5×10^9^/L and hemoglobin > 70g/L without transfusion support) post-HSCT in the presence of CDC. Primary PT was defined as the failure to achieve platelet engraftment by +60 days post-HSCT and secondary PT as the fulfillment of the criteria after initial platelet engraftment [33]. Engraftment was marked by ANC > 0.5×10^9^/L for 3 consecutive days without G-CSF administration, platelet > 20×10^9^/L for 7 consecutive days without platelet transfusion, and hemoglobin > 70 g/L without red blood cell transfusion. In contrast, graft rejection, defined as never having achieved engraftment with mixed chimerism or complete recipient chimerism. Any measurable level of residual disease (MRD) as assessed by multiparameter flow cytometry and/or polymerase chain reaction was considered positive at the time of transplant [34, 35]. Relapse, NRM, LFS, and OS were defined as previously described [30, 35]. aGVHD were graded according to the literature [36]. AEs were graded based on the National Cancer Institute Common Terminology Criteria for Adverse Events (CTCAE version 4.0) with the exception of hematologic AEs. Sinusoidal obstruction syndrome (SOS) was diagnosed according to the criteria stated by Jones et al. an onset before day 21 of hyperbilirubinemia and two of the following, weight gain >5%, tender hepatomegaly, and ascites [37].

### Statistical analysis

This trial was designed to test the hypothesis that NAC prophylaxis was superior to nonprophylaxis in terms of PGF or PT. The sample size was calculated based on the incidence of primary or secondary PGF or PT at +60 days, which was approximately 30% in the AL patients with BM EC < 0.1% pre-haplo-HSCT without NAC prophylaxis [5, 31, 33, 38]. To identify a 20% absolute decrease in the incidence of PGF or PT with NAC prophylaxis, a minimum of 120 patients (80 in the study group and 40 in the control group) was required to provide the study with a one-sided significance level of 0.025 and a power of 80%. After adjusting for a 10% dropout, the total planned sample size was 130 patients. The sample size calculation was performed using PASS software (version 11.0).

The chi-square and Mann-Whitney *U* tests were performed for categorical variables and continuous variables, respectively. Cumulative incidences of PGF or PT, myeloid and platelet engraftment, relapse, NRM, and GVHD were calculated by accounting for competing risks using the Fine and Gray model [39]. OS and LFS were estimated by the Kaplan-Meier method and compared by the log-rank test. The corresponding hazard ratio (HR) and 95% CI were estimated using the Cox proportional hazard model. All variables in Table 1 were included in the univariable analysis. Only variables with *P* < 0.15 were included in the multivariable analysis. All statistical tests were two-tailed with a significance level of 0.05 except for the superiority hypothesis. SPSS 20.0 (SPSS Inc., Chicago, IL, USA) and R version 3.3.0 (R Development Core Team, Vienna, Austria) were used for data analysis.

**Table 1:** Patient and donor characteristics

Characteristics	Low-risk arm (group C)	High-risk arm	
		NAC prophylaxis cohort (group A)	Control cohort (group B)
Number of patients	105	80	40
Median age (range), years	31 (15–55)	31 (15–53)	30 (15–48)
Male gender, *N* (%)	61 (58.1)	41 (51.3)	27 (67.5)
Diagnosis, *N* (%)			
AML	57 (54.3)	41 (51.2)	22 (55.0)
refined disease risk index			
Low (favorable cytogenetics, any CR)	3 (5.3)	6 (14.6)	0
Intermediate (intermediate cytogenetics, any)CR)	30 (52.6)	21 (51.2)	12 (54.5)
High (adverse cytogenetics, any CR)	24 (42.1)	14 (34.1)	10 (45.5)
Remission status			
First CR (CR1)	51 (90.5)	37 (90.2)	22 (100)
Second CR (CR2)	6 (9.5)	4 (9.8)	0
ALL	48 (45.7)	39 (48.8)	18 (45.0)
refined disease risk index			
Intermediate (CR1)	47 (97.9)	37 (94.9)	17 (94.4)
High (CR2)	1 (2.1)	2 (5.1)	1(5.6)
Philadelphia positive	15 (31.3)	11 (28.2)	6 (33.3)
Measurable residual disease before transplant, *N* (%)			
Negative	65 (61.9)	53 (66.2)	23 (57.5)
Positive	40 (38.1)	27 (33.8)	17 (42.5)
Median time from diagnosis to HSCT(range), months	5 (3–24)	5 (2–42)	5 (3–12)
Median donor age(range), years	40 (10–64)	45 (10–66)	37 (8–61)
HLA-A, B, DR mismatched grafts, *N* (%)			
1	5 (4.8)	2 (2.4)	1 (2.5)
2	21 (20.0)	15 (18.8)	5 (12.5)
3	79 (75.2)	63 (78.8)	34 (85.0)
Donor-recipient gender matched, *N* (%)			
Male-male	43 (41.0)	31 (38.8)	20 (50.0)
Male-female	29 (27.6)	30 (37.5)	11 (27.5)
Female-male	18 (17.1)	10 (12.5)	7 (17.5)
Female-female	15 (14.3)	9 (11.2)	2 (5.0)
Donor-recipient relationship, *N* (%)			
Father-child	37 (35.2)	42 (52.5)	18 (45.0)
Mother-child	11 (10.5)	4 (5.0)	2 (5.0)
Sibling-sibling	23 (21.9)	12 (15.0)	7 (17.5)
Child-parent	30 (28.6)	19 (23.8)	13 (32.5)
Collateral relatives	4 (3.8)	3 (3.8)	0
ABO matched grafts, *N* (%)			
Matched	50 (47.6)	43 (53.8)	23 (57.5)
Major mismatch	22 (21.0)	17 (21.3)	5 (12.5)
Minor mismatch	25 (23.8)	15 (18.8)	10 (25.0)
Bi-directional mismatch	8 (7.6)	5 (6.3)	2 (5.0)
Median chemo cycles pre-HSCT (range)	3 (2–9)	3 (2–12)	3 (2–14)
≥2 Induction cycles to achieve CR, *N* (%)	18 (17.1)	16 (20.0)	9 (22.5)
Median CD34^+^ cells, 10^6^/kg (range)	2.86 (0.77–14.10)	3.15 (0.49–9.42)	3.31 (0.92–7.45)

*Abbreviations: NAC N*-acetyl-L-cysteine, *AML* Acute myeloid leukemia, *ALL* Acute lymphoblastic leukemia, *HLA* Human leukocyte antigen, *HSCT* Hematopoietic stem cell transplantation, *chemo* chemotherapy, *CR* Complete remission

## Results

### Study population

Between April 18, 2019, and June 24, 2021, 254 patients with AL scheduled to undergo first HSCT from haploidentical donors were screened. 130 were identified as high-risk (EC < 0.1%) for developing PGF or PT, and 124 were identified as low-risk (EC ≥ 0.1%). After screening, 10 patients with EC < 0.1% were excluded before randomization due to leukemia relapse (diagnosed by the same BM sample for EC detection), donor change (strongly positive donor-specific antibodies, donor decline), or contraindication to HSCT (organ failure diagnosed during the overall medical examination following BM EC detection before HSCT). Therefore, 120 of the 130 high-risk patients were randomly assigned at a 2:1 ratio to either receive (group A; *N*=80) or not receive (group B, *N*=40) NAC prophylaxis. Nineteen of the 124 low-risk patients (group C) were also excluded from data analysis due to leukemia relapse, donor change, contraindication to HSCT, or hesitation to undergo HSCT. The study flow diagram is shown in Fig. 1, and 120 randomized patients were included in the efficacy and safety analysis. Except for the BM EC percentage, the three groups (A, B, C) had balanced patient, donor and transplant characteristics including CD34^+^ cells infused, disease risk index [40], and MRD status before transplant (Table 1).

### NAC prophylaxis significantly reduced PGF or PT

**Fig. 2:**
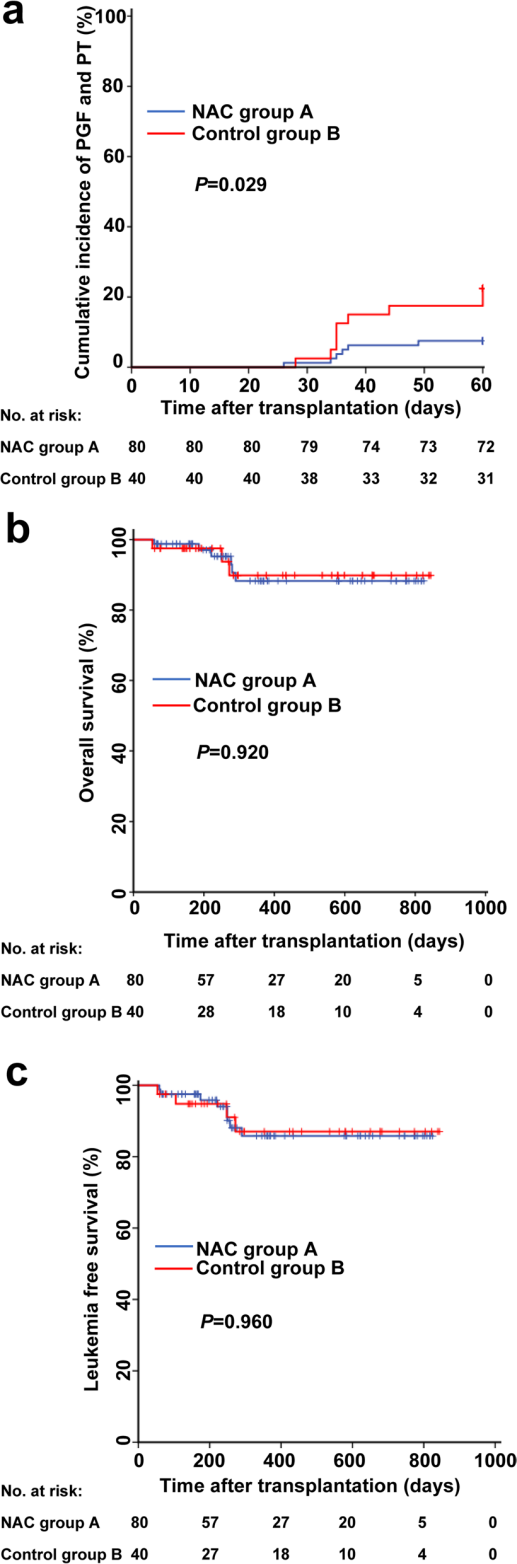
Cumulative incidence of PGF or PT (**a**), overall survival (**b**), and leukemia-free survival (**c**)

The +60 days cumulative incidence of PGF or PT was 7.5% (95% CI, 1.7 to13.3%) in group A compared to 22.5% (95% CI, 9.1 to 35.9%; *P =* 0.021) in group B and 11.4% (95% CI, 5.2 to17.6%; *P =* 0.373) in group C (Fig. 2a). Within +60 days, there were 27 PGF or PT including 1 primary PGF, 16 secondary PGF, 3 primary PT, and 7 secondary PT. Sensitivity analyses excluding subjects receiving haplo-HSCT using rituximab for desensitization with a positive donor-specific antibody indicated that there were 7.8% (95% CI, 1.8 to13.8%) with PGF or PT in group A (*N* = 77) compared to 23.1% (95% CI, 9.5 to 36.7%) in group B (*N* = 39; *P =* 0.023) and 9.9% (95% CI, 3.9 to 15.9%) in group C (*N* = 101; *P =* 0.627). The median time to platelet engraftment is shown in Table 2. Univariable analysis of PGF or PT is shown in Table 3. Results of an exploratory post hoc subgroup analysis are shown in Table 4.

**Table 2:** Transplant outcomes

Parameter	Low-risk arm (group C, *N*=105)	High-risk arm	
		NAC prophylaxis cohort (group A, *N*=80)	High-risk control cohort (group B, ***N***=40)
Median days of neutrophil engraftment (range)	13 (11–42)	12 (11–14)	12 (11–14)
Median days of platelet engraftment (range)	13 (9–168)	13 (9–217)	13 (10–219)
Acute GVHD at day 100, %(95% CI)	17.4(10.0–29.8)	26.2(16.6–35.8)	25.3(11.3–39.3)
CMV reactivation at day 100, %(95% CI)	84.8(78.0–91.6)	87.5(80.1–94.9)	85.0(73.2–95.8)
EBV reactivation at day 100, % (95% CI)	15.4( 8.0–22.8)	11.3(3.9–18.7)	12.7(2.1–23.3)
1-year incidence of relapse, %(95% CI)	6.7(1.0–12.3)	5.2(0–11.2)	6.5(0–15.5)
1-year incidence of NRM, %(95% CI)	3.2(0–6.6)	9.1(1.1 17.1)	6.4(0–15.4)
1-year probability of LFS, %(95% CI)	90.1(83.3–96.9)	85.8(77.5 95.0)	87.0(74.6–99.4)
1-year probability of OS, %(95% CI)	95.7(91.5–100)	88.3(79.1–97.5)	89.8(78.4–100)

There were no statistically significant differences in any of these transplant outcomes between the three groups

*Abbreviations: NAC N*-acetyl-L-cysteine, *GVHD* graft-versus-host disease, *CI* confidential interval, *CMV* cytomegalovirus, *EBV* Epstein-Barr virus, *NRM* non-relapse mortality, *LFS* leukemia-free survival, *OS* overall survival

**Table 3:** Univariable and multivariable analyses for the risk factors of PGF or PT and survival post-transplantation

Parameters	PGF/PT		Overall survival		Leukemia-free survival	
	Univariable HR (95%CI) ***P***	Multivariable HR (95%CI) ***P***	Univariable HR (95%CI) ***P***	Multivariable HR (95%CI) ***P***	Univariable HR (95%CI) ***P***	Multivariable HR (95%CI) ***P***
**Patient gender** **male vs. female**	0.796 (0.374–1.694) 0.554	-	0.459 (0.150–1.402) 0.171	-	0.751 (0.313–1.805) 0.523	-
**Patient age** **< 30y vs ≥ 30y (median)**	1.136 (0.534–2.416) 0.741	-	0.083 (0.0011–0.638) *0.017	0.091 (0.012–0.708) *0.022	0.249 (0.083–0.746) *0.013	0.321 (0.085–1.213) 0.094
**Diagnosis** **AML vs. ALL**	0.804 (0.378–1.711) 0.572	-	0.736 (0.247–2.190) 0.581	-	1.030 (0.427–2.486) 0.947	-
**Donor age** **< 45y vs ≥ 45y (median)**	0.635 (0.297–1.357) 0.242	-	1.411 (0.461–4.316) 0.546	-	1.92 (0.56–6.57) 0.300	-
**HLA disparity** **3/6vs.4**–**5/6**	0.661 (0.289–1.511) 0.327	-	0.545 (0.168–1.733) 0.314	-	0.985 (0.329–2.949) 0.979	-
**Donor gender** **male vs. female**	0.732 (0.329–1.629) 0.444	-	0.431 (0.145–1.283) 0.130	0.594 (0198–1.780) 0.352	0.545 (0.222–1.334) 0.184	
**Relationship** **Parents vs. other**	1.279 (0.593–2.756) 0.530	-	0.533 (0.174–1.630) 0.270	-	0.362 (0.139–0.943) *0.038	0.645 (0.202–2.059) 0.459
**ABO blood type** **other vs. major mismatch**	0.679 (0.287–1.606) 0.378		0.366 (0.048–2.819) 0.335	-	0.224 (0.030–1.673) 0.145	0.205 (0.027–1.534) 0.123
**CD34+ cell infused** **≥ median vs.< median**	0.429 (0.182–1.016) 0.054	0.400 (0.163–0.984) *0.046	0.575 (0.177–1.868) 0.357	-	0.692 (0.276–1.735) 0.433	-
**Chemo cycles before HSCT** **<4 vs. ≥4 cycles**	2.068 (0.905–4.724) 0.085	1.851 (0.798–4.293) 0.151	0.805 (0.270–2.399) .696	-	1.423 (0.581–3.487) 0.440	-
**Cycles to achieve CR** **<2 vs. ≥2 cycles**	1.014 (1.014–4.724) 0.978		0.484 (0.149–1.572) 0.227	-	0.661 (0.2401–1.821) 0.424	-
**NAC prophylaxis** **NAC vs. high-risk control**	*0.025 0.317 (0.113–0.890) *0.021	*0.029 0.274 (0.096–0.777) *0.015	0.463 1.064 (0.266–4.257) 0.930	-	0.792 1.028 (0.309–3.414) 0.964	-
**NAC vs. low-risk control**	0.643 (0.242–1.715) 0.373	0.725 (0.270–1.945) 0.523	2.150 (0.606–7.621) 0.236	-	1.378 (0.517–3.672) 0.521	-

*Abbreviations: AML* acute myeloid leukemia, *ALL* acute lymphoblastic leukemia, *HLA* human leukocyte antigen, *HR* hazard ratios, *CI* confidence interval, *HSCT* hematopoietic stem cell transplantation, *chemo* chemotherapy, *CR* complete remission, *NAC N*-acetyl-L-cysteine; *P< 0.05

**Table 4:** Subgroup analyses for PGF or PT post-transplantation

Characteristics	Low-risk arm (group C), %(95% CI)	High-risk arm		
		NAC prophylaxis cohort (group A), %(95% CI)	Control cohort (group B), %(95% CI)	***P*** value
Diagnosis				
AML	12.2(3.6–20.8)	4.8(0–11.4)	18.2(1.4–35.0)	0.088
ALL	10.4(1.8–19.0)	10.2(0.6–19.8)	27.8(7.0–48.6)	0.106
Donor age				
<45	9.5(2.3–16.7)	5.2(0–12.4)	18.2(1.6–34.8)	0.102
≥45	14.2(3.6–24.8)	9.5(0.7–18.3)	27.8(7.0–48.6)	0.083
Donor-recipient relationship				
Parents	9.6(1.4–17.8)	10.2(1.5–18.9)	30.0(9.2–50.8)	0.050
Others	13.2(4.0–22.4)	3.2(0–9.4)	15.0(0–31.2)	0.128
Pre-HSCT chemo cycles				
<4	18.3(8.3–28.3)	9.7(0.5–18.9)	19.0(1.6–36.4)	0.331
≥4	2.2(0–6.6)	5.1(0–12.0)	26.3(5.5–47.1)	0.022
Cycles to achieve CR <2 vs. ≥2 cycles				
<2	11.5(5.0–18.0)	7.8(1.2–14.4)	22.6(7.4–37.8)	0.048
≥2	11.1(0–26.3)	6.3(0–18.7)	22.2(0–51.8)	0.234
Infused CD34^+^ cells, 10^6^/kg				
<3	13.6(0–22.0)	12.2(2.0–22.4)	31.6(9.8–53.3)	0.084
≥3	7.7(0–16.1)	2.6(0–5.3)	14.3(0–29.9)	0.084

*Abbreviations: NAC N*-acetyl-L-cysteine, *AML* acute myeloid leukemia, *ALL* acute lymphoblastic leukemia, *CI* confidence interval, *HSCT* hematopoietic stem cell transplantation, *chemo* chemotherapy, *CR* complete remission

Multivariable analysis revealed that NAC prophylaxis (*P* = 0.029; NAC prophylaxis vs. high-risk control: HR 0.274, 95% CI, 0.096 to 0.777; *P* = 0.015; NAC prophylaxis vs. low-risk control: HR 0.725, 95% CI, 0.270 to1.945; *P* = 0.523) and CD34^+^ cell count higher than the median (HR 0.400, 95% CI, 0.163 to 0.984; *P* = 0.046) were associated with lower incidence of PGF or PT (Table 3).

### NAC prophylaxis did not affect neutrophil engraftment, GVHD, or virus infection

Neutrophil engraftment by +28 days was achieved in all of the patients except one in group C. There was no primary graft rejection. The median time to neutrophil engraftment did not differ between the three groups (Table 2). The 100-day cumulative incidence of aGVHD, CMV and EBV infection was also similar (Table 2).

### NAC prophylaxis did not affect NRM, CIR, or survival

With a median follow-up of 347 days (range, 60–844 days) (Table 1) post-HSCT, the 1-year probabilities relapse, NRM, LFS, and OS were similar among the three groups (Table 2 and Fig. 2b, c). At the last follow-up, 212 patients survived, and 13 died after a median of 187 days (range, 54–290 days) post-HSCT. Causes of death are shown in Table 5.

**Table 5:** Causes of death in the three groups

Causes of death	NAC group A (***N***=80)	High-risk control group B (***N***=40)	Low-risk control group C (***N***=105)
Total	6(8%)	3(8%)	4(4%)
Relapse	1(1%)	1(3%)	2(2%)
GVHD	1(1%)	0	0
Infections	3(4%)	1(3%)	1(1%)
Other	1(1%)	1(3%)	1(1%)

*Abbreviations: GVHD* Graft-versus-host disease

### Adverse events

AEs from enrollment to +60 days post-HSCT are shown in Table 6. Four patients died of AEs (three infections in group A and one infection in group B). All the grade 3 to 5 AEs were nontreatment-related. Since NAC is known to reduce the hepatotoxicity including SOS incidence and reduces Bu/Cy side effects [41, 42], liver function test values for both groups A and B are shown in Table 7. Liver enzymes and bilirubin values were most highly elevated in the high-risk control group B compared to NAC group A after BU conditioning, at +21 days, +60 days post-HSCT, although it did not reach statistical significance. None of the patients developed SOS.

**Table 6:** Adverse effects

	NAC group A (***N***=80)				High-risk control group B (***N***=40)			
	Grades 1–2	Grade 3	Grade 4	Grade 5	Grades 1–2	Grade 3	Grade 4	Grade 5
Skin^**a**^	25(31)	0	0	0	8(20)	0	0	0
Gastrointestinal^**a**^	54(67)	16(20)	0	0	30(70)	7(18)	0	0
Hepatobiliary/pancreatic^**a**^	14(17)	5(6)	0	0	5(13)	1(3)	0	0
Cardiac	12(15)	1(1)	0	0	7(18)	0	0	0
Renal/genitourinary	8(10)	0	0	0	6(15)	0	0	0
Infections^**b**^	5(6)	16(20)	0	3(4)	2(5)	8(20)	1(3)	1(3)
Nervous system disorders	2(3)	0	0	0	1(3)	0	0	0

Grade 1–2 adverse events in more than 10 of patients and all grade 3–5 adverse events were recorded from enrollment to 60 days post-transplantation

^a^Excluded the patients with GVHD

^b^Excluded the patients with cytomegalovirusviremia and Epstein-Barr virus viremia

**Table 7:** Liver enzymes and bilirubin

Parameters	Alanine aminotransferase (ALT) Median (range), U/l		Aspartate aminotransferase (AST) Median (range), U/l		Bilirubin Median (range), μmol/l	
	NAC group A (***N***=80)	High-risk Control group B (***N***=40)	NAC group A (***N***=80)	High-risk Control group B (***N***=40)	NAC group A (***N***=80)	High-risk Control group B (***N***=40)
**Before busulfan**	19.5 (2.0–152.0)	22.0 (5.0–82.0)	18.0 (9.0–75.0)	19.5 (9.0–40.0)	9.7 (3.2–30.8)	10.5 (4.4–22.7)
**After busulfan**	15.5 (4.0–151.0)	19.5 (6.0–91.0)	17.0 (11.0–83.0)	17.0 (10.0–43.0)	9.5 (4.0–33.1)	9.8 (4.6–19.0)
**Day 21 post transplant**	34.5 (7.0–926.0)	33.0 (8.0–300.0)	25.0 (11.0–317.0)	27.0 (8.0–158.0)	10.7 (4.0–27.1)	12.1 (3.8–20.2)
**Day 60 post transplant**	19.0 (4.0–254.0)	22.0 (8.0–156.0)	23.0 (10.0–312.0)	25.0 (14.0–76.0)	11.6 (6.4–116.7)	11.8 (6.3–41.1)

*Abbreviations: NAC N-*acetyl-L-cysteine, *AST* aspartate aminotransferase (normal range <40U/l), *ALT* alanine aminotransferase (normal range <40U/l), bilirubin (normal range <26μmol/l)

### Prophylactic NAC improved the quantity of BM ECs and their ROS levels in the EC < 0.1% group

**Fig. 3:**
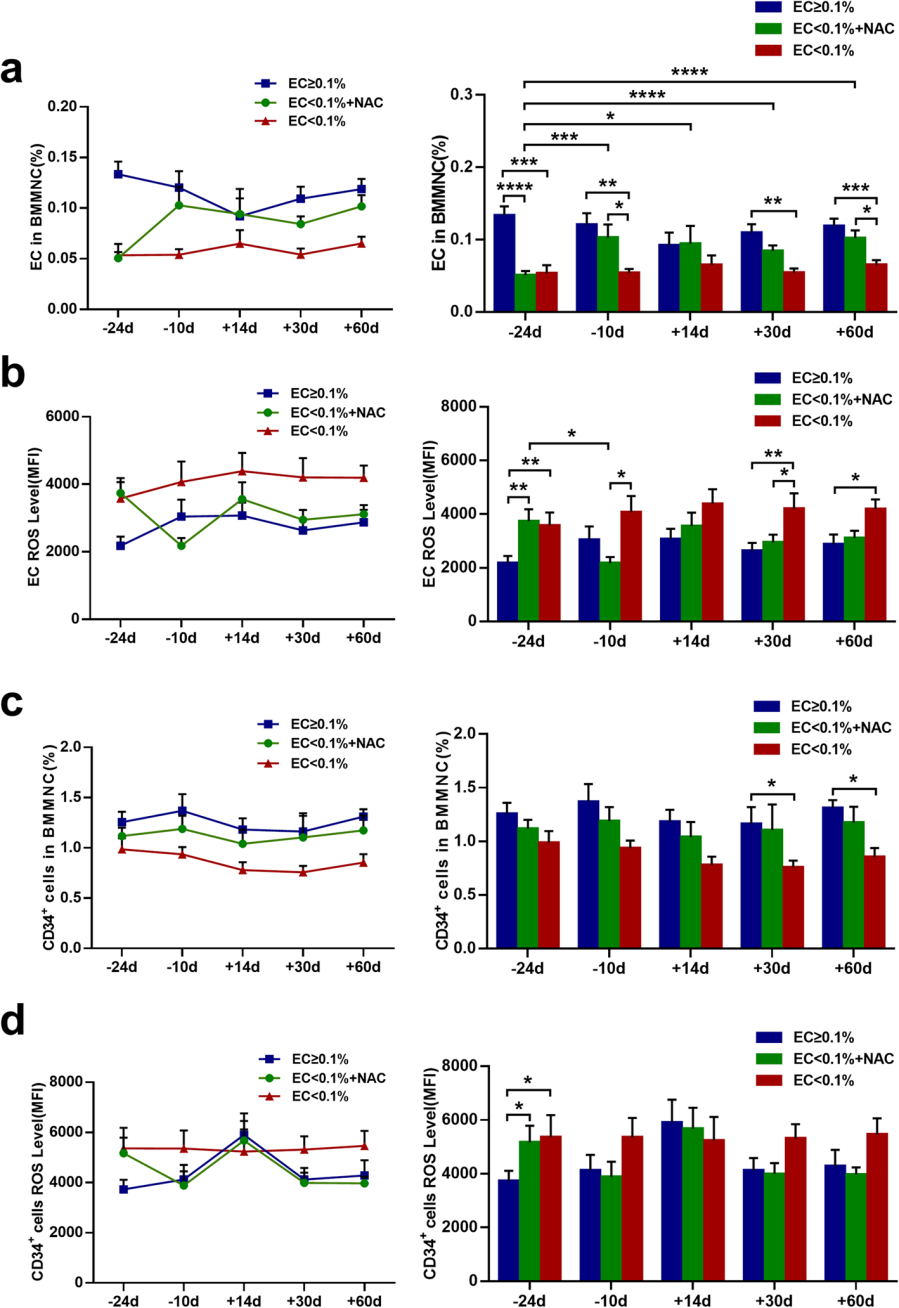
Prophylactic NAC improved BM ECs and CD34^+^ cells in EC<0.1% group post-HSCT. The dynamic reconstitution (left panel) and statistical analysis (right panel) of **a** BM EC percentage, **b** EC ROS level, **c** CD34^+^ cell percentage, and **d** CD34^+^ cell ROS level were analyzed by flow cytometry among the three groups before randomization (−24 days), at the time of conditioning initiation (−10 days), and +14, +30, +60 days post-HSCT. The data are expressed as the mean and SEM. *P* ≤ 0.05 was considered statistically significant and values are provided in the figure (*P ≤ 0.05, ** *P* ≤ 0.01, *** *P* ≤ 0.001, **** *P* ≤ 0.0001)

To evaluate whether prophylactic NAC could improve the impaired BM ECs and hematopoiesis post-HSCT, the quantity and function of BM ECs and CD34^+^ cells were investigated kinetically before randomization (−24 days) at conditioning initiation (−10 days), +14 days, +30 days, and +60 days post-HSCT. The percentage of BM ECs in group C was significantly higher than the baseline level in group A and group B. Prophylactic NAC gradually improved the percentage (Fig. 3a; −10 days: 0.10±0.02 *vs*. 0.05±0.005; *P* = 0.02; +60 days: 0.10±0.01 *vs*. 0.07±0.007; *P* = 0.03) and functions of BM ECs including double-positive staining (Additional file 1: Fig. S1a; +60 days: 78.17±6.65 *vs.* 36.33±5.25; *P* = 0.001), the abilities of migration (Additional file 1: Fig. S1b; −10 days:162.17±19.56 *vs.* 92.5±4.26; *P* < 0.0001; +14 days: 121.33±16.16 *vs.* 68.67±13.39; *P* = 0.002; +60 days: 105.33±3.04 *vs.* 44.17±10.71; *P* = 0.0004), and tube formation (Additional file 1: Fig. S1c; +14 days: 579.72±49.14 *vs.* 174.97±51.22; *P* = 0.0005; +60 days: 599.32±34.23 *vs.* 240.57±42.44; *P* = 0.002) of BM ECs in group A compared with those in group B.

Elevated baseline ROS level in BM ECs was observed in group A (Fig. 3b; 3738.91±444.29 *vs*. 2179.90±265.73; *P* = 0.002) and group B (Fig. 3b; 3575.17±489.31 *vs*. 2179.90±265.73; *P* = 0.005) compared with group C before randomization. NAC prophylaxis significantly reduced the ROS level of BM ECs (Fig. 3b; 2178.92±227.67 *vs*. 3738.91±444.29; *P* = 0.02) at – 10 days compared to their baseline level in group A. In contrast, significantly higher ROS level of BM ECs was found at −10 days (Fig. 3b; 4069.43±604.72 *vs*. 2178.92±227.67; *P* = 0.01) and +30 days (Fig. 3b; 4204.17±569.79 *vs*. 2948.94±287.54; *P* = 0.03), and +60 days (Fig. 3b; 4192.74±360.1 *vs*. 3112.75±271.26; *P* = 0.07) in group B than in group A.

After NAC prophylaxis in group A, the percentage of BM ECs was significantly increased (Fig. 3a; 0.10±0.02 vs. 0.05±0.006; *P* = 0.0008). Moreover, NAC prophylaxis gradually improved the percentage and functions including double-positive staining (Additional file 1: Fig. S1a; −10 days:75.67±16.99 *vs*. 95.5±6.52; *P* = 0.19; +14 days: 58.67±6.61 *vs*. 84±15.20; *P* = 0.1; +30 days: 59.5±4.19 *vs*. 76.17±11.82; *P* = 0.27), the abilities of migration (Additional file 1: Fig. S1b; −10 days: 162.17±19.56 *vs*. 162.67±9.45; *P*=0.98; +14 days: 121.33±16.16 *vs*. 145.67±17.01; *P* = 0.2; +30 days: 82±7.66 *vs*. 108.33±10.26; *P* = 0.16) and tube formation (Additional file 1: Fig. S1c; −10 days: 470.22±122.76 *vs*. 912.1±65.52; *P* = 0.01; +14 days: 579.72±49.14 *vs*. 490.88±57.05; *P* = 0.56; +30 days: 377.58±63.07 *vs*. 781.9±185.53; *P* = 0.01) of BM ECs from patients in group A to the similar degree as those of group C. In contrast, no significant improvement in BM ECs was found in group B.

### Prophylactic NAC improved the quantity of BM CD34^+^ cells and their ROS levels in the EC < 0.1% group

Consistent with the improvement of BM ECs, NAC prophylaxis gradually increased the percentage of BM CD34^+^ cells in group A to similar levels to those in group C at +14 days (Fig. 3c; 1.04±0.14 *vs*. 1.18±0.11; *P* = 0.59), +30 days (Fig. 3c; 1.1±0.24 *vs*. 1.16±0.16; *P* = 0.76), and +60 days (Fig. 3c; 1.17±0.15 *vs*. 1.31±0.07; *P* = 0.47), which were better than those in group B. The ROS levels of CD34^+^ cells in group A were gradually reduced to similar levels to those in group C at +30 days (Fig. 3d; 3987.06±406.87 *vs*. 4125.72±458.56; *P* = 0.85), +60 days (Fig. 3d; 3971.03±267.72 *vs*. 4285.54±607.1; *P* = 0.67), which were remarkably lower than those in group B.

Taken together, our data indicate that oral NAC prophylaxis could improve impaired BM EC reconstitution and therefore better support donor CD34^+^ cell engraftment post-HSCT.

## Discussion

The current study is a phase 3, open-label randomized trial to demonstrate that NAC prophylaxis could promote hematopoietic reconstitution by improving the quantity and function of BM ECs after allo-HSCT and thereby reduce the incidence of PGF or PT in BM EC < 0.1% high-risk patients.

In this study, our results achieved the expected primary objective of testing for a reduction in the incidence of PGF or PT for the NAC and high-risk control groups (7.5% vs. 22.5%) while NAC prophylaxis obtained similar outcomes to those in low-risk group C. These results are consistent with our previous single-arm reports. In addition, the current randomized trial results further validated that BM EC is a reliable marker for predicting PGF or PT and that EC-directed NAC prophylaxis could offset the detrimental EC effect on poor hematopoietic reconstitution after allo-HSCT [5].

Unraveling how to improve dysfunctional BM ECs to enhance hematopoiesis will be of great importance to guide the establishment of new approaches. Recently, Hu et al. reported that multiple antioxidants, such as NAC, sulforaphane, and resveratrol, could alleviate the damage of radiation-induced bystander effects to human HSCs mainly through regulating their oxidative stress [43]. However, to our knowledge, the current RCT is the first to establish a novel BM microenvironment-directed antioxidant therapy to promote hematopoiesis in HSCT patients based on pathogenesis.

Tolerability is another issue of concern apart from efficacy. The overall grade 3 to 5 AEs within +60 days post-HSCT were similar between groups A and B and were nontreatment-related. In addition, NAC use did not affect GVHD or virus infection despite reducing PGF/PT, maybe partly due to the insufficient power to detect the difference for the second endpoints when considering the negative impact of PGF/PT on GVHD and viral infection, and also the intensified immune suppression itself having great effect on GVHD and viral infection.

We acknowledge the limitation of the relatively short follow-up and insufficient power to detect the difference for the second endpoints including GVHD, viral infection, and other transplant outcomes with respect to the clinical benefit. Nevertheless, the concerns about bleeding and infection risk caused by poor hematopoietic reconstitution might be ameliorated. Furthermore, NAC is reported to be a potential prophylactic treatment for hepatotoxicity during BU conditioning. With this regard, the lower dose and the oral administration of NAC in our study as compared to higher i.v. dose in previous reports might attribute to less striking effect [41, 42]. Longer follow-up and quality-of-life assessments as well as further studies with larger sample size are needed to explore additional clinical benefit.

## Conclusions

In summary, a phase 3, open-label randomized trial confirmed that BM EC<0.1% pre-HSCT can identify high-risk patients for the occurrence of PGF or PT post-HSCT. Convenient oral NAC prophylaxis was safe and effective in preventing the occurrence of poor hematopoietic function by repairing impaired BM ECs. Therefore, our data indicate that improvement of the BM microenvironment may offer a potential pathogenesis-oriented therapeutic approach for poor hematopoietic function for future validation.

## Supplementary Information

**Additional file 1: Figure S1.** Prophylactic NAC improved function of ECs in EC<0.1% group to similar levels in EC>0.1% group.

**Additional file 2.** Study protocol and statistical analysis plan.

## Data Availability

The data that support the findings of this study are available upon reasonable request from the corresponding author.

## Abbreviations

allo-HSCT: Allogeneic hematopoietic stem cell transplantation
AL: Acute leukemia
Ara-C: Cytarabine
ATG: Antithymocyte globulin
AEs: Adverse events
BM: Bone marrow
BMMNCs: BM mononuclear cells
Bu: Busulfan
CR: Complete remission
Cy: Cyclophosphamide
CsA: Cyclosporin A
CIR: Cumulative incidences of leukemia relapse
CDC: Complete donor chimerism
DiI-AcLDL: Dil-acetylated low-density lipoprotein
ECs: Endothelial cells
FITC-UEA-1: Fluorescein isothiocyanate–labeled Ulex Europaeus Agglutinin-1
GVHD: Graft-versus-host disease
HSCs: Hematopoietic stem cells
haplo-HSCT: Haploidentical-HSCT
LFS: Leukemia-free survival
MMF: Mycophenolate
MTX: Methotrexate
MRD: Measurable residual disease
NAC: *N*-Acetyl-L-cysteine
NRM: Nonrelapse mortality
OS: Overall survival
PGF: Poor graft function
PT: Prolonged isolated thrombocytopenia
ROS: Reactive oxygen species
SOS: Sinusoidal obstruction syndrome
TID: Three times daily
TPO: Thrombopoietin
ULN: Upper limit of normal
VEGFR2: Vascular endothelial growth factor receptor 2
