# Supplementary material for: Prophylactic NAC promoted hematopoietic reconstitution by improving endothelial cells after haploidentical HSCT: a phase 3, open-label randomized trial
Source: BMC Med. 2022 Apr 27;20:140. doi: 10.1186/s12916-022-02338-9 (PMC9044574; doi:10.1186/s12916-022-02338-9)
Supplement: Supplementary file 1 — Additional file 1: Figure S1. Prophylactic NAC improved function of ECs in EC<0.1% group to similar levels in EC>0.1% group. [file 12916_2022_2338_MOESM1_ESM.pdf]

Prophylactic NAC Promoted Hematopoietic Reconstitution by Improving Endothelial Cells after haploidentical HSCT：a Phase 3, Open-label Randomized Trial

Authors: Yu Wang[1#], Yuan Kong[1#], Hong-Yan Zhao[1], Yuan-Yuan Zhang[1], Ya-Zhe Wang[1], Lan-Ping Xu[1], Xiao-Hui Zhang[1], Kai-Yan Liu[1], Xiao-Jun Huang[1,2]*

[#]Yu Wang and Yuan Kong contributed equally to this study.

Correspondence: Xiao-Jun Huang MD,

[1]Peking University People's Hospital, Peking University Institute of Hematology, Beijing Key Laboratory of Hematopoietic Stem Cell Transplantation, Collaborative Innovation Center of Hematology, Peking University, Beijing, 100044, China;

[2]Peking-Tsinghua Center for Life Sciences, Academy for Advanced Interdisciplinary Studies, Peking University, Beijing, 100871, China.

T 8610 88326006

F 8610 88324577

E huangxiaojun@bjmu.edu.cn

Fig. S1. Prophylactic NAC improved function of ECs in EC<0.1% group to similar levels in EC>0.1% group.

Fig. S1

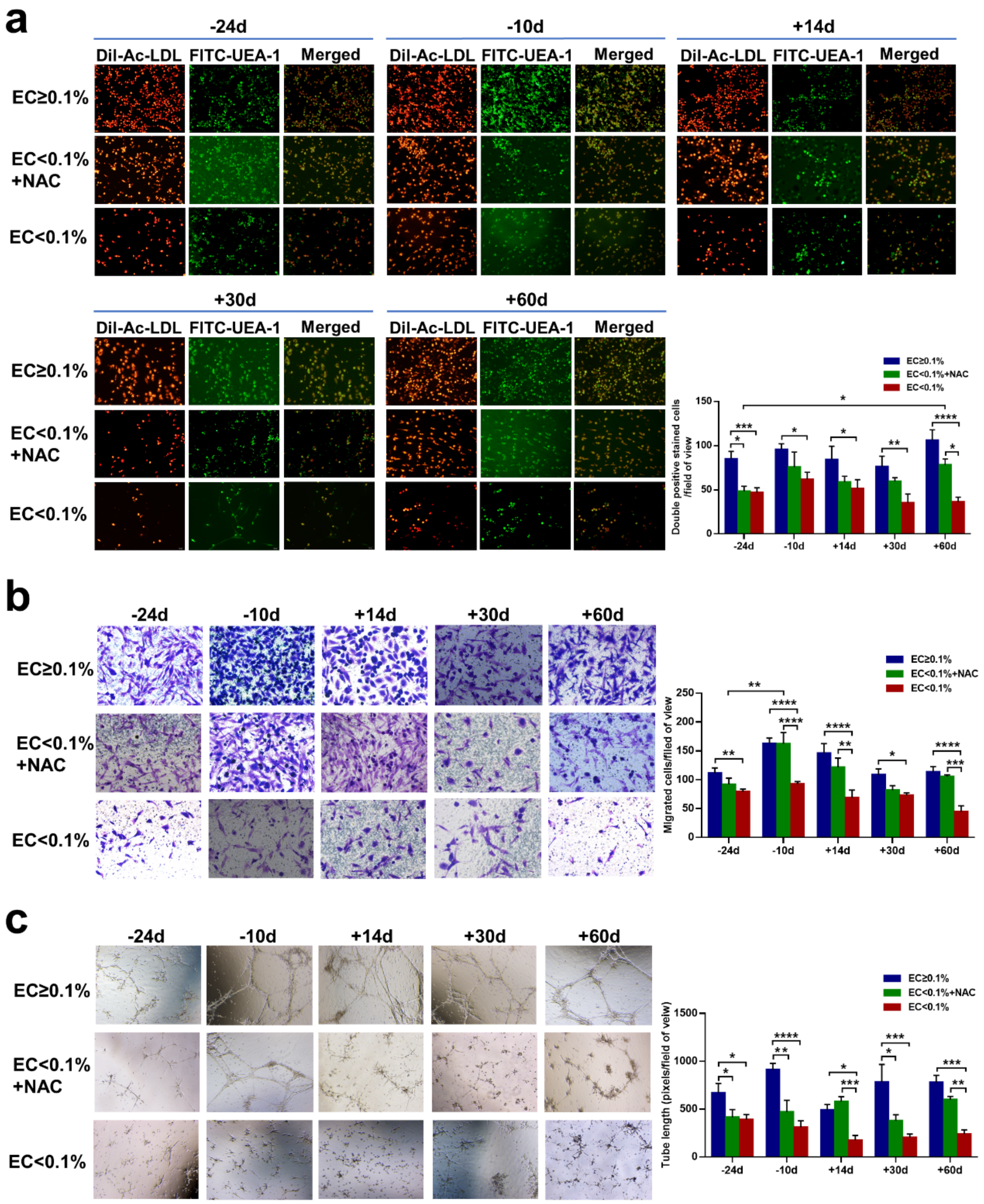

Fig. S1. Prophylactic NAC improved function of ECs in EC<0.1% group to similar levels in EC>0.1% group. (a) Representative images of typical BM ECs characterized by double-positive staining (merged in yellow) of both DiI-AcLDL(red) and FITC-UEA-1(green) (original magnification×10) and

quantification of BM double-positive staining cells (second row right panel) per field of view were analyzed among the three groups before randomization(-24d), at the time of conditioning initiation(-10d), and +14, +30, +60d post-HSCT. **(b)**Representative images (left panel, original magnification×10) and quantification (right panel) of transwell migration assays of cultivated BM ECs per field of view were analyzed among the three groups. **(c)**Representative images (left panel, original magnification×10) and quantification (right panel) of tube formation (pixels of tubes per field of view) assays of cultivated BM ECs were analyzed among the three groups. The data are expressed as the mean and SEM. $P \leq 0.05$ was considered statistically significant and values are provided in the figure (*$P \leq 0.05$, ** $P \leq 0.01$, *** $P \leq 0.001$, **** $P \leq 0.0001$).
