# Supplementary material for: Prophylactic NAC promoted hematopoietic reconstitution by improving endothelial cells after haploidentical HSCT: a phase 3, open-label randomized trial
Source: BMC Med. 2022 Apr 27;20:140. doi: 10.1186/s12916-022-02338-9 (PMC9044574; doi:10.1186/s12916-022-02338-9)
Supplement: Supplementary file 2 — Additional file 2. Study protocol and statistical analysis plan. [file 12916_2022_2338_MOESM2_ESM.pdf]

CLINICAL STUDY PROTOCOL

Protocol Title: Risk Stratification-directed N-acetyl-L-cysteine for Prevention of poor hematopoietic reconstitution After Unmanipulated Haploidentical stem cell Transplantation——an open-label, Randomized, Controlled, Clinical Trial

Protocol Number: 2019PHB-047

Study Drug: N-acetyl-L-cysteine (NAC)

Study Phase: 3

Indication: acute leukemia in complete remission undergoing haploidentical hematopoietic stem cell transplantation

Applicant Institution: Peking University People's Hospital
Principal institution: Peking University People's Hospital

Version: 2.0
Date: 20181128

Remark: the primary version of this protocol was in Chinese. We have translated it into English.

INVESTIGATOR'S STATEMENT

I have received and completely reviewed the following protocol (2019PHB-047, Protocol Version 2.0, dated 28 November 2018), including all appendices:

As Principal Investigator, I understand and agree to conduct this clinical study as described and will comply with the ethical and regulatory considerations delineated herein.

Study Title

Risk Stratification-directed N-acetyl-L-cysteine for Prevention of poor hematopoietic reconstitution After Unmanipulated Haploidentical stem cell Transplantation——an open-label, Randomized, Controlled, Clinical Trial

Principal Investigator Signature and Contact Information

**Principal Investigator (print)**	**Xiao-Jun Huang**
**Principal Investigator (signature)**	
**Date of Signature**	**2018-11-28**
**Institution/Affiliation**	**Peking University People's Hospital**
**City, Province, Country**	**Beijing China**

Study Synopsis

**Study title**	Risk Stratification-directed N-acetyl-L-cysteine for Prevention of poor hematopoietic reconstitution After Unmanipulated Haploidentical stem cell Transplantation——an open-label, Randomized, Controlled, Clinical Trial
**Protocol number**	2019PHB-047
**Indication**	acute leukemia in complete remission undergoing haploidentical hematopoietic stem cell transplantation
**Study phase**	3
**Study applicant**	Peking University People's Hospital
**Study center**	Peking University People's Hospital
**Number of subjects planned**	Approximately 120 subjects (80 in NAC group and 40 in control group) will be randomized.
**Study duration**	Estimated to be 2 years
**Objectives**	**Primary objective:**<br>To compare the incidence of PGF and PT, which was assessed at +2M post-HSCT in patients with acute leukemia undergoing haplo-HSCT who receive NAC prophylaxis versus non-prophylaxis peri-transplantation.<br>**Secondary objectives:**<br>To compare cumulative incidences of leukemia relapse (CIR), GvHD, non-relapse mortality (NRM), leukemia-free survival (LFS), overall survival (OS), and adverse effects (AEs) in patients with acute

	leukemia undergoing haplo-HSCT who receive NAC prophylaxis versus non-prophylaxis peri-transplantation.
**Study design**	This is a prospective, open-label, randomized, phase 3 study comparing NAC prophylaxis with non-prophylaxis peri-transplantation for patients with leukemia undergoing haplo-HSCT. Approximately 120 subjects will be randomized in a 2:1 ratio to receive NAC prophylaxis (80 subjects) or non-prophylaxis (40 subjects) post-transplantation.Randomization is done with permuted blocks (block size four), and implemented through an interactive web-based response system.<br><br>**NAC Group:** NAC prophylaxis (NAC is administrated at 14 days before conditioning and continued until day +60 post-transplantation).<br><br>**Control Group:** Non- prophylaxis (Neither NAC nor thrombopoietin (TPO)/thrombopoietin receptor agonists (TPO-RAs) is used until day +60 post-transplantation).<br><br>BM assessment was performed before randomization, at the time of conditioning initiation, +14d, +28d, +60d (including BM smear and EC detection till +60d), +90d post-HSCT, and every three months thereafter until the study was completed (+1Y post-HSCT).<br><br>All subjects will be followed for safety and tolerability within 60 days post-transplantation. With the exception of hematologic AEs, all AEs are graded according to CTCAE version 4.0
**Inclusion criteria**	Subjects eligible for enrolment in this study must meet all of the following criteria:<br><br>1. Patients with acute leukemia (AL) undergoing first haplo-HSCT<br><br>2. Age 15 to 60 years old with Eastern Cooperative Oncology Group (ECOG) performance status 0-2

	3. complete remission (CR) before HSCT (CR must be confirmed by BM analysis within 3 days before randomization)<br>● CR is defined as bone marrow (BM) blasts <5%; absence of circulating blasts and blasts with Auer rods; absence of extramedullary disease; absolute neutrophil count (ANC) $\geq 1{\cdot}0\times10^{9}$/L and platelet count (PLT) $\geq 100\times10^{9}$/L. Hematopoietic recovery within 60 days post-transplantation<br>4. bone marrow (BM) Endothelial Cell (EC) <0.1% at screening before conditioning<br>5. Sign informed consent form, have the ability to comply with study and follow-up procedures
**Exclusion criteria**	Subjects meeting any of the following criteria are ineligible for this study:<br>1. Acute leukemia not in remission<br>2. hypersensitivity to NAC or history of bronchial asthma<br>3. Life expectancy less than 30 days post-transplantation<br>4. uncontrolled infections pre-transplantation<br>5. Cardiac dysfunction (particularly congestive heart failure, unstable coronary artery disease and serious cardiac ventricular arrhythmias requiring antiarrhythmic therapy)<br>6. Respiratory failure (PaO2 ≤60mmHg)<br>7. Hepatic abnormalities (total bilirubin ≥2 times the upper limit of normal [ULN], alanine aminotransferase or aspartate aminotransferase ≥2 times the ULN)<br>8. Renal dysfunction (creatinine ≥1.5 times the ULN or

	creatinine clearance rate < 30 mL/min)<br>9. ECOG performance status 3, 4 or 5<br>10. With any conditions not suitable for the trial (investigators' decision)
**Study treatment**	**NAC prophylaxis**<br>After enrollment, patients in the study group (NAC arm) were scheduled for NAC prophylaxis. If the patients met the inclusion criteria on day 14 before conditioning, they received NAC from day 14 before conditioning until day +60 post-HSCT. The initial dose of NAC was 400mg orally three times daily (TID). In cases of grade 3 or worse AEs (not including hematologic recovery), dose modifications including dose reductions or interruptions were permitted at the physician's discretion. After the resolution of AEs, the dose was re-escalated from to 400mg TID.<br>**Non-prophylaxis**<br>For the participants in the control group (non-prophylaxis arm), neither NAC nor thrombopoietin (TPO)/thrombopoietin receptor agonists (TPO-RAs) were administered before day +60 post-HSCT.
**Sample size determination**	This trial was designed to test the hypothesis that NAC prophylaxis peri-HSCT was superior to non-prophylaxis in terms of PGF and PT. The sample size was calculated based on the primary endpoint, the +2M incidence of PGF and PT, which was approximately 30% in the AL patients with BM EC< 0.1% pre-haplo-HSCT without NAC prophylaxis.20 To identify a 20% absolute decrease in the +2M incidence of PGF and PT with NAC prophylaxis, a minimum of 120 patients (80 in the study group and 40 in the control group) was required to provide the study with a one-sided significance level of

	0.025 and a power of 80%. After adjusting for a 10% dropout, the total planned sample size was 130 patients.
**Statistical analysis**	Statistical analysis is performed based on the intent-to-treat (ITT) population, which includes all randomized subjects.<br>**Primary Efficacy Analysis:**<br>The primary endpoint was the incidence of PGF and PT, which was assessed at +2M post-HSCT.<br>● PGF was defined as the presence of 2 or 3 cytopenic counts (ANC≤0.5×109/L, platelet≤20×109/L, or hemoglobin≤70 g/L) for at least 3 consecutive days beyond day +28 post-HSCT with a transfusion requirement, related with hypoplastic-aplastic BM, in the presence of complete donor chimerism (CDC).<br>● PT was defined as platelet count less than 20×109/L or a dependence on platelet transfusion with the engraftment of other cell lines(ANC>0.5×109/L and hemoglobin>70 g/L without transfusion support) beyond day +60 post-HSCT in the presence of CDC<br>**Secondary Efficacy Analysis:**<br>The secondary endpoints of OS and LFS are estimated using the Kaplan-Meier method and compared using the log-rank test.<br>● OS is defined as the time from transplantation until death from any cause.<br>● LFS is defined as survival in continuous CR without relapse, and refer to the time from transplantation until relapse or death from any cause.<br>**Safety Analysis:**<br>Safety and tolerability will be assessed by incidence and severity of AEs and changes from baseline of all relevant parameters, including laboratory test values, physical examination, vital signs, and ECOG

	performance scores. With the exception of hematologic AEs, all AEs are graded according to CTCAE version 4.0. All subjects will be monitored for AEs within 60 days post-transplantation.

TABLE OF CONTENTS

PAGE

Clinical Study Protocol 1
Investigator's Statement 2
Study Synopsis 3
Table of Contents 9
Abbreviations 11
1. Introduction 13
2. Study objectives 14
  2.1 Primary Objective 14
  2.2 Secondary Objectives 14
3. Study Design 14
4. Subject Selection Criteria 16
  4.1 Subject Selection Criteria 16
    4.1.1 Number of Subjects 16
    4.1.2 Inclusion Criteria 17
    4.1.3 Exclusion Criteria 17
  4.2. Withdrawal Criteria 18
5. Study Procedures 19
  5.1 Screening 19
  5.2 Treatment Allocation and Blinding 19
  5.3 Study Treatment 19
    5.3.1 NAC Group (NAC prophylaxis) 19
    5.3.2 Control Group (non-prophylaxis) 20
  5.4 Follow-up 20
6. Efficacy Assessments 20
  6.1 Definitions 20
  6.2 Primary Efficacy Endpoint 21
  6.3 Secondary Efficacy Endpoints 21
  6.4 Schedule and methods of Efficacy Assessments 21
7. Safety Evaluation 22
  7.1 Medical History 22
  7.2 Vital Signs and Physical Examination 22
  7.3 Clinical Symptoms 23
  7.4 Clinical Laboratory Evaluations 23
8. Adverse Events and Serious Adverse Events (SAEs) 24
  8.1 Definitions 24
    8.1.1 Adverse Events 24
    8.1.2 Serious Adverse Events 24
  8.2 Assessment of Severity 25
  8.3 Assessment of Causality 26
  8.4 Recording and Reporting AEs and SAEs 26
9. Rules of Withdrawal 27
  9.1 Subjects Withdraw from the Study 27

9.2 Premature Termination of the Study 27
10. Rules of Follow-Up 28
10.1 Follow-up Period 28
10.2 Visit Scheduling 28
10.3 Contents 28
11. Data Analysis and Statistical Considerations 28
11.1 Hypotheses 28
11.2 Study Design Considerations 29
11.2.1 Sample Size Assumptions 29
11.2.2 Primary Efficacy Endpoint 29
11.2.3 Secondary Efficacy Endpoints 30
11.3 Data Analysis Considerations 30
11.3.1 Analysis Population 30
11.3.2 Analysis Plan 30
11.3.2.1 Baseline Data 30
11.3.2.2 Analysis of Efficacy 30
11.3.2.3 Analysis of Safety 31
12. Materials for the Study 31
13. Ethical Considerations 31
13.1 Responsibility of Investigators 31
13.2 Informed Consent Process 31
13.3 Good Clinical Practice 32
13.4 Protection of Subjects' Personal Data 32
14. Administrative Requirements 32
15. References 33
16. Appendices 35
16.1 Appendix 1 Diagnosis and Classification of aGVHD and cGVHD 35
16.2 Appendix 2 ECOG Performance Satus 40

Abbreviations

AEs	Adverse events
allo-HSCT	Allogeneic hematopoietic stem cell transplantation
ALT	Alanine aminotransferase
ANCs	Neutrophils
AST	Aspartate aminotransferase
BM	Bone marrow
BMMNCs	Bone marrow mononuclear cells
BSA	Body surface area
CFU-GEMM	Colony forming unit-granulocyte; erythroid; macrophage and monocyte
CIR	Cumulative incidences of relapse
CR	Complete remission
CRFs	Case Report Forms
CTCAE	Common Terminology Criteria for Adverse Events
CMV	Cytomegalovirus
LFS	leukemia free survival
DSMC	Data and safety monitoring committee
ECs	Endothelial cells
ECOG	Eastern Cooperative Oncology Group
EPCs	Endothelial progenitor cells

GVHD	Graft-versus-host disease
haplo-HSCT	Haploidentical allogeneic hematopoietic stem cell transplantation
HSC	Hematopoietic stem cell
IRB	Institution review board
MKs	Megakaryocytes
MSCs	Mesenchymal stem cells
NAC	N-acetyl-L-cysteine
NRM	Non-relapse mortality
OS	Overall survival
PGF	Poor graft function
PLT	Platelets
PT	Prolonged isolated thrombocytopenia
rhG-CSF	Recombinant human granulocyte colony-stimulating factor
ROS	Reactive oxygen species
SAEs	Serious adverse events
TBL	Total bilirubin
TPO	Thrombopoietin
ULN	Upper limits of normal
VEGFR	Vascular endothelial growth factor receptor

1. Introduction

Allogeneic hematopoietic stem cell transplantation (allo-HSCT) is an effective treatment of malignant hematopoietic diseases. However, poor hematopoietic reconstitution including poor graft function (PGF) and prolonged isolated thrombocytopenia (PT), remains a life-threatening complication after allo-HSCT. Especially with the increasing use of haploidentical allo-HSCT (haplo-HSCT) in the past ten years, PGF and PT have become growing obstacles contributing to high morbidity and mortality after allo-HSCT. Due to the limited mechanism studies, the clinical management of PGF and PT is challenging.

Our recent prospective case-control studies reported that the reduced and dysfunctional Bone marrow (BM) endothelial cells (ECs) after allo-HSCT are involved in the pathogenesis of PGF and PT. Moreover, in vitro treatment with N-acetyl-L-cysteine (NAC) could enhance the defective hematopoietic stem cell (HSC) function through repairing the dysfunctional BM ECs of PGF and PT patients[1-3]. We performed a small-scale pilot cohort study and saw encouraging clinical results that oral administration with NAC could partially repair the dysfunctional BM ECs and improve megakaryocytopoiesis in PT patients[3], which suggests that NAC is a promising drug in PT patients after allo-HSCT. In addition, our prior prospective trial suggests that BM ECs<0.1% pre-HSCT is the risk factor for occurrence of the 2M PGF and PT following allo-HSCT.

Therefore, we designed the study with acute leukemia patients who will be scheduled to receive haplo-HSCT. The percentages and ROS levels of BM ECs and CD34+ cells will be evaluated at -14 day before-HSCT. The BM ECs <0.1% pre-HSCT patients, who are willing to accept the oral treatment with NAC from -14 day pre-HSCT to +2 months post-HSCT continuously, will be enrolled in the RCT study. Exclusive criteria are bronchila asthma and NAC allergy. The BM ECs ≥0.1% pre-HSCT patients will receive haplo-HSCT without prophylactic intervention with NAC. The aim of the trial is to evaluate the efficacy of the prophylactic administration of NAC in acute leukemia patients with complete remission pre- and post-allotransplant on the occurrence of PGF and PT after haplo-HSCT.

2. Study objectives

2.1 Primary Objective

The primary objective of this study is to compare the incidence of PGF and PT, which was assessed at +2M post-HSCT in patients with acute leukemia undergoing haplo-HSCT who receive NAC prophylaxis versus non-prophylaxis peri-transplantation.

2.2 Secondary Objectives

The secondary objectives of this study are to compare cumulative incidences of leukemia relapse (CIR), GvHD, non-relapse mortality (NRM), leukemia-free survival (LFS), overall survival (OS), and adverse effects (AEs) in patients with acute leukemia undergoing haplo-HSCT who receive NAC prophylaxis versus non-prophylaxis peri-transplantation.

3. Study Design

This is a prospective, open-label, randomized, phase 3 study of comparison of NAC prophylaxis versus non-prophylaxis peri-transplantation in patients with leukemia

undergoing haplo-HSCT. The study design is illustrated in Figure 1.

Subjects with leukemia in CR receiving first haplo-HSCT will be screened for eligibility. Medical history evaluation, vital sign, physical examination, Eastern Cooperative Oncology Group (ECOG) performance status, blood and urine sampling for laboratory tests, electrocardiogram, chest imaging examination as well as bone marrow (BM) assessment will be performed to determine study eligibility, all of which must be performed ≤3 days prior to randomization. Eligible subjects will be randomized in a 2:1 ratio to receive NAC prophylaxis versus non-prophylaxis peri-transplantation. Randomization is performed with randomization codes generated by a computer-generated randomization system.

Based on the randomization and assignment, the subjects will receive NAC prophylaxis versus non-prophylaxis peri-transplantation. For subjects in the experimental intervention arm (NAC group), if the patients met the inclusion criteria on day 14 before conditioning, they received NAC from day 14 before conditioning until day +60 post-HSCT. The initial dose of NAC was 400mg orally three times daily (TID). In cases of grade 3 or worse AEs (not including hematologic recovery), dose modifications including dose reductions or interruptions were permitted at the physician's discretion. After the resolution of AEs, the dose was re-escalated from to 400mg TID. For subjects in the no intervention arm (non-prophylaxis, control group), neither NAC nor thrombopoietin (TPO)/thrombopoietin receptor agonists (TPO-RAs) were administered before day +60 post-HSCT. No crossover between the two groups before +60d will be allowed.

BM assessment was performed before randomization, at the time of conditioning initiation, +14d, +28d, +60d (including BM smear and EC detection till +60d), +90d post-HSCT, and every three months thereafter until the study was completed (+1Y post-HSCT).

All subjects will be followed for safety and tolerability within 60 days post-transplantation. With the exception of hematologic AEs, all AEs are graded according to CTCAE version 4.0

Figure 1 Study Schema

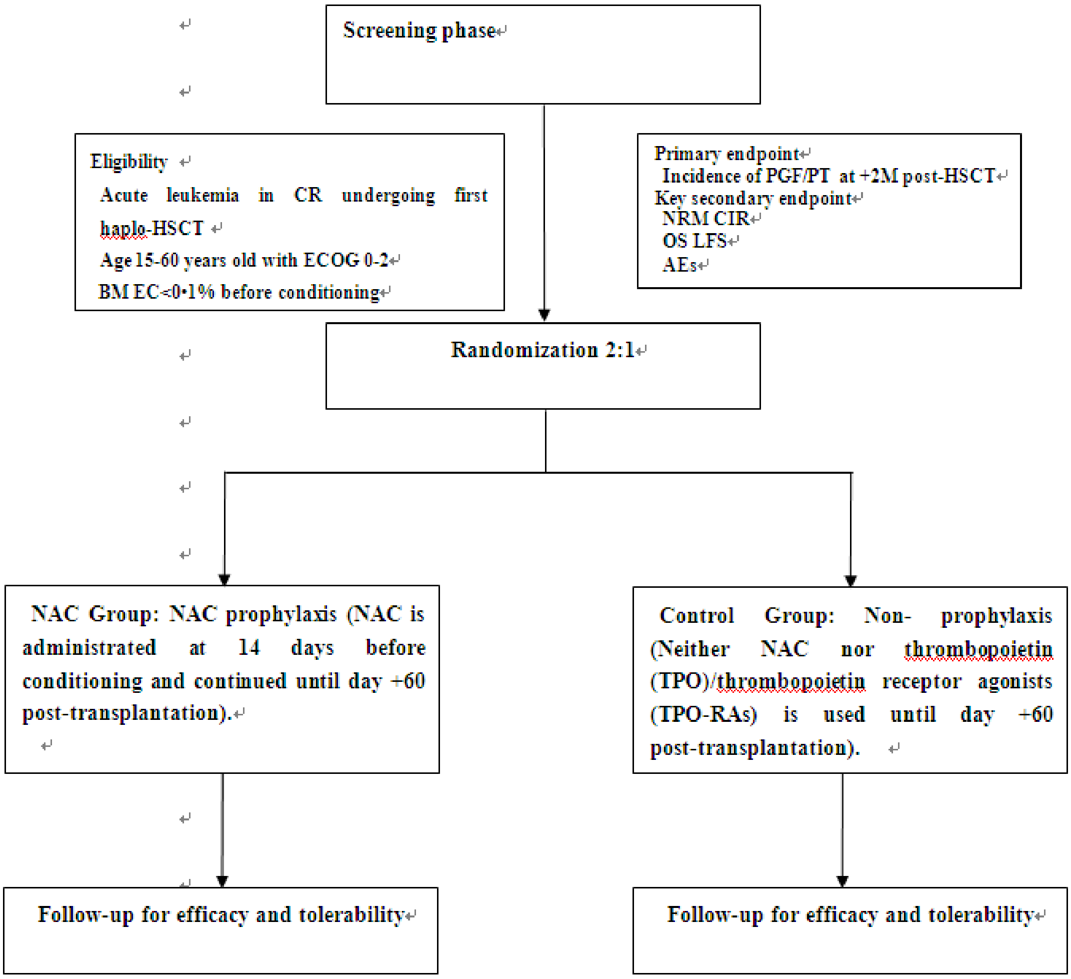

CR= complete remission; haplo-HSCT= haploidentical hematopoietic stem cell transplantation; ECOG= Eastern Cooperative Oncology Group; NRM= non-relapse mortality; OS= overall survival; LFS= leukemia-free survival; AEs= adverse effects.

4. Subject Selection Criteria

4.1 Subject Selection Criteria

4.1.1 Number of Subjects

Approximately 120 subjects will be randomized to NAC or control group (80 subjects in NAC group and 40 in control group).

4.1.2 Inclusion Criteria

Subjects eligible for enrolment in this study must meet all of the following criteria:

1. Patients with acute leukemia (AL) undergoing first haplo-HSCT

2. Age 15 to 60 years old with Eastern Cooperative Oncology Group (ECOG) performance status 0-2

3. complete remission (CR) before HSCT (CR must be confirmed by BM analysis within 3 days before randomization)

- CR is defined as one marrow (BM) blasts <5%; absence of circulating blasts and blasts with Auer rods; absence of extramedullary disease; absolute neutrophil count (ANC) $\geq 1\cdot 0\times 10^9$/L and platelet count (PLT) $\geq 100\times 10^9$/L.

4. bone marrow (BM) Endothelial Cell (EC) <0.1% at screening before conditioning

5. Sign informed consent form, have the ability to comply with study and follow-up procedures

4.1.3 Exclusion Criteria

Subjects meeting any of the following criteria are ineligible for this study:

1. Acute leukemia not in remission

2. hypersensitivity to NAC or history of bronchial asthma

3. Life expectancy less than 30 days post-transplantation

4. uncontrolled infections pre-transplantation

5. Cardiac dysfunction (particularly congestive heart failure, unstable coronary artery disease and serious cardiac ventricular arrhythmias requiring antiarrhythmic therapy)

6. Respiratory failure ($PaO_2 \leq 60$mmHg)

7. Hepatic abnormalities (total bilirubin ≥2 times the upper limit of normal [ULN], alanine aminotransferase or aspartate aminotransferase ≥2 times the ULN)

8. Renal dysfunction (creatinine ≥1.5 times the ULN or creatinine clearance rate < 30 mL/min)

9. ECOG performance status 3, 4 or 5

10. With any conditions not suitable for the trial (investigators' decision)

4.2. Withdrawal Criteria

Subjects are free to withdraw consent and discontinue participation in the study at any time and without prejudice to future treatment. A subject's participation in the study may be discontinued at any time at the investigator's discretion. Justifiable reasons for a subject to be withdrawn from the study include:

1. Inability to fully comply with the study protocol
2. Initiation of alternative treatment of TPO/TRO-RA within +60d post-HSCT
3. Unacceptable toxicity
4. Best interest of the subject based upon the investigator’s discretion
5. At the request of the study subject at any time and for any reason

Subjects will be followed up unless the informed consent is withdrawn. The reason for withdrawal from study participation and the date must be documented in the case report form (CRF). The investigator must complete the last visit, including vital signs, physical examination, laboratory tests, disease status and AE assessment, all of which must be documented in the CRF.

5. Study Procedures

5.1 Screening

Subjects with acute leukemia receiving first haplo-HSCT in CR will be screened for eligibility. Medical history evaluation, vital sign, physical examination, ECOG performance status, blood and urine sampling for laboratory tests, electrocardiogram, chest imaging examination as well as BM assessment including smear and EC detection will be performed to determine study eligibility, all of which must be performed ≤3 days prior to randomization.

5.2 Treatment Allocation and Blinding

This is an open-label study. Neither subjects nor investigators will be blinded to treatment. Upon completion of all the required screening assessments, eligible subjects will be randomized at 2:1 ratio to receive NAC prophylaxis or non-prophylaxis peri-transplantation. Randomization is done with permuted blocks (block size four), and implemented through an interactive web-based response system.

5.3 Study Treatment

5.3.1 NAC Group (NAC prophylaxis)

After enrollment, patients in the study group (NAC arm) were scheduled for NAC prophylaxis. If the patients met the inclusion criteria on day 14 before conditioning, they received NAC from day 14 before conditioning until day +60 post-HSCT. The initial dose of NAC was 400mg orally three times daily (TID). In cases of grade 3 or worse AEs (not including hematologic recovery), dose modifications including dose reductions or interruptions were permitted at the physician's discretion. After the resolution of AEs, the dose was re-escalated from to 400mg TID.

5.3.2 Control Group (non-prophylaxis)

Neither NAC nor thrombopoietin (TPO)/thrombopoietin receptor agonists (TPO-RAs) were administered before day +60 post-HSCT.

5.4 Follow-up

BM assessment was performed before randomization, at the time of conditioning initiation, +14d, +28d, +60d (including BM smear and EC detection till +60d), +90d post-HSCT, and every three months thereafter until the study was completed (+1Y post-HSCT).

All subjects will be followed for safety and tolerability within 60 days post-transplantation. With the exception of hematologic AEs, all AEs are graded according to CTCAE version 4.0.

6. Efficacy Assessments

6.1 Definitions

- PGF was defined as the presence of 2 or 3 cytopenic counts (ANC≤0.5×109/L, platelet≤20×109/L, or hemoglobin≤70 g/L) for at least 3 consecutive days beyond day +28 post-HSCT with a transfusion requirement, related with hypoplastic-aplastic BM, in the presence of complete donor chimerism (CDC)
- PT was defined as platelet count less than 20×109/L or a dependence on platelet transfusion with the engraftment of other cell lines(ANC>0.5×109/L and hemoglobin>70 g/L without transfusion support) beyond day +60 post-HSCT in the presence of CDC
- Good graft function(GGF) was defined as a persistent successful engraftment, as marked by ANC>0.5×109/L for 3 consecutive days without granulocyte colony-stimulatingfactor (G-CSF) administration, platelet >20×10^9/L for 7

consecutive days without platelet transfusion, and hemoglobin >70g/L without red blood cell transfusion, beyond day +28 post-HSCT

- Relapse is defined as reappearance of leukemic blasts in the peripheral blood or ≥ 5% blasts in the BM as pirate or biopsy not attributable to any other cause or reappearance or new appearance of extramedullary leukemia.
- CR is defined as BM blasts <5%; absence of circulating blasts and blasts with Auer rods; absence of extramedullary disease; ANC $\geq 1\cdot 0\times 10^9$/L and PLT $\geq 100\times 10^9$/L.

6.2 Primary Efficacy Endpoint

- The incidence of PGF and PT, which was assessed at +2M post-HSCT.

6.3 Secondary Efficacy Endpoints

- OS is defined as the time from transplantation until death from any cause.
- LFS is defined as survival in continuous CR without relapse, and refer to the time from transplantation until relapse or death from any cause.

6.4 Schedule and methods of Efficacy Assessments

Engraftment assessment including routine blood and BM assessment will be regularly performed peri-transplantation. Routine blood is monitored before randomization, twice a week before conditioning, daily from conditioning until engraftment, then twice a week until +30d post-HSCT, once a week from the 2nd to 3rd month post HSCT, and then once every two weeks until the study is completed. BM assessment was performed before randomization, at the time of conditioning initiation, +14d, +28d, +60d (including BM smear and EC detection till +60d), +90d post-HSCT, and every three months thereafter until the study was completed (+1Y post-HSCT). All subjects who complete treatment or withdraw from the study must receive efficacy assessment.

7. Safety Evaluation

All subjects enrolled in the study will be evaluable for safety and tolerability. Safety and tolerability will be assessed with vital signs, physical examination, clinical symptoms, and clinical laboratory evaluations (hematology, serum chemistry, urinalysis, electrocardiogram, and chest imaging examination). With the exception of hematologic AEs, all AEs will be evaluated within 60 days post-transplantation according to CTCAE version 4.0.

7.1 Medical History

Each subject's medical history must be obtained at screening. Information on any prior or existing medical conditions will be recorded on the appropriate CRF.

7.2 Vital Signs and Physical Examination

Vital signs and results of physical examination must be documented before randomization, once a week for the first month after enrollment, once every two weeks from engraftment until 60 days post-transplantation. The next 8 items must be performed:

- Physical examination
- Heart rate
- Blood pressure
- Body temperature
- Rate of respiration
- Body weight
- ECOG performance status
- Signs of infection

7.3 Clinical Symptoms

During the study, the patients' clinical symptoms must be documented. The clinical symptoms may be associated with the administration of NAC reported previously, including allergy such as rash, hand-foot-skin reaction, dermatitis, erythema, flushing, pruritus, dry skin, or bronchospasm; gastrointestinal reaction such as diarrhea, nausea, vomiting, or anorexia.

7.4 Clinical Laboratory Evaluations

Before initiation of the study, the monitors will document the normal range of each test in every involved laboratory. During the study, the next items must be performed:

- Routine blood: white cell counts, neutrophil cell counts, hemoglobin, and platelet counts
- Hepatic function: total bilirubin (both direct bilirubin and indirect bilirubin must be documented when the total bilirubin elevates), ALT, AST, lactic dehydrogenase, alkaline phosphatase, albumin and total protein
- Renal function: serum creatinine, urea nitrogen and uric acid
- Other biochemical indicators: amylase and lipase
- Electrolytes: sodium, potassium, calcium and magnesium
- Coagulation function: prothrombin time, prothrombin time-international normalized ratio, activated partial thromboplastin time and fibrinogen
- Urinalysis: protein, glucose and erythrocyte
- Electrocardiogram
- Chest imaging examination

8 Adverse Events and Serious Adverse Events (SAEs)

The investigator is responsible for detecting, documenting and reporting events that meet the definition of an AE or SAE.

8.1. Definitions

8.1.1 Adverse Events

An AE is any untoward medical occurrence in a subject of a clinical investigation, which does not necessarily have a causal relationship to the medicinal product. Therefore, an AE can be any unfavorable and unintended sign, including an abnormal laboratory finding, symptom, or disease (new or exacerbated), whether or not it is considered to be related to the product. This definition includes any newly occurring event or previous condition that has increased in severity or frequency since the administration of the product. However, hematologic recovery or death due to PGF/PT should not be recorded as AEs.

8.1.2 Serious Adverse Events

A serious adverse event is any untoward medical occurrence that, at any dose:

- Results in death
- Is life-threatening
- Requires hospitalization or prolongation of existing hospitalization - ie, the AE requires at least a 24-hour inpatient hospitalization or prolongs a hospitalization beyond the expected length of stay.
  Hospitalization or prolongation of existing hospitalization for social reasons will not be reported as an SAE.
- Results in disability/incapacity
- Congenital anomaly/ birth defect
- Important medical event

Medical or scientific judgment should be exercised in deciding whether SAE reporting is appropriate in other situations. An important medical event is an event that may not result in death, be life-threatening, or require hospitalization, but is clearly of major clinical significance. The AE may jeopardize the subject or require intervention to prevent a serious outcome.

8.2 Assessment of Severity

With the exception of hematologic AEs, all AEs are graded according to CTCAE version 4.0. When CTCAE version 4.0 criteria do not apply, severity will be defined according to the following criteria:

**Severity**	**Description**
Grade 1- Mild	Asymptomatic or mild symptoms; clinical or diagnostic observations only; intervention not indicated
Grade 2- Moderate	Minimal, local or noninvasive intervention indicated; limiting age-appropriate instrumental activities of daily living (ADL)
Grade 3- Severe	Medically significant but not immediately life threatening; hospitalization or prolongation of hospitalization indicated; disabling; limiting self-case ADL
Grade 4- Life-threatening	Life-threatening consequences; urgent intervention indicated
Grade 5- Death	Death

8.3 Assessment of Causality

The investigator must determine the relationship of each AE and SAE to study treatment. Relationship of an AE or SAE to study treatment will be defined according to the following criteria:

- Definite: There is a clear temporal relationship to study treatment, with no other possible cause.
- Possible: A temporal relationship to study treatment is not clear, and alternative etiologies are possible.
- Not related: There is no temporal relationship to study treatment, and/or there is evidence of an alternative cause such as a concurrent medication or illness.

8.4 Recording and Reporting AEs and SAEs

All AEs and SAEs must be recorded in the appropriate CRF, whether or not they are associated to be causally related to study treatment. Each SAE must be reported promptly on the Serious Adverse Event Report Form, and submitted to the Independent Ethics Committee within 24 hours by the investigator. The information recorded on the Serious Adverse Event Report Form will include at least the following: subject number, identity of the event, study drug name and dose, investigator's assessment of the event's severity and relationship to study treatment, and investigator's name and signature. Clinical monitors must collect and verify detailed information of AEs and SAEs when examining original medical records. All AEs and SAEs should be followed up until resolved.

9. Rules of Withdrawal

9.1. Subjects Withdraw from the Study

Subjects can withdraw from the study at any time for any reason without impact on the investigator’s right to treat disease for subjects. Based upon the interest of subjects, the investigator has the right to request subjects to withdraw from the study for any

reason including concomitant disease, AEs or treatment failure. The core group of clinical study reserves the right to request subjects to withdraw from the study for deviation(s) from the protocol, administrative reasons, or other effective or ethical reasons.

The last assessment for subjects must be performed and documented in the CRF regardless of the time and reason for withdrawal. The reason for withdrawal from study participation must be documented in the CRF. All documents related to subjects should be completed. Despite withdrawal from the study, those subjects should be followed up and documented about their diseases until withdrawal of informed consents.

For subjects who withdraw from the study due to concomitant diseases or AEs, the details must be documented in the CRF with other appropriate and valuable data attached.

9.2. Premature Termination of the Study

Reasons for premature termination of the study include external events, repetition of SAEs, growing incidence of treatment-related death and slow enrolment in the study. All subjects will be informed of premature termination of the study by written consents. Any subjects who decide to discontinue participating in the study must report to the principal investigator.

10. Rules of Follow-Up

10.1 Follow-up Period

Starting from randomization.

10.2 Visit Scheduling

Every week for the first three months after enrollment, and then every month until the study is completed.

10. 3 Contents

The contents of every follow-up visit include complaints of subjects, vital signs, physical examination, clinical symptoms and clinical laboratory evaluations (hematology, serum chemistry, urinalysis, electrocardiogram, chest imaging examination, and BM assessment). All of the results must be documented in the original medical record.

11. Data Analysis and Statistical Considerations

11.1. Hypotheses

The primary endpoint is the +2m incidence of PGF and PT post-transplantation. The null and alternative hypotheses are designed with the goal of demonstrating the superiority of NAC prophylaxis over non-prophylaxis post-transplantation with respect to PGF and PT. Superiority will be determined using the following hypothesis:

H0: incidence of PGF and PT with NAC prophylaxis ≥ incidence of PGF and PT with non-prophylaxis post-transplantation

H1: incidence of PGF and PT with NAC prophylaxis < incidence of PGF and PT with non-prophylaxis post-transplantation

11.2 Study Design Considerations

This prospective, open-label, randomized, phase 3 study compares the efficacy of NAC prophylaxis and non-prophylaxis peri-transplantation in acute leukemia undergoing haplo-HSCT. The primary outcome is the +2m incidence of PGF and PT post-transplantation, and the study is designed to determine if NAC prophylaxis is superior to non-prophylaxis peri-transplantation in the study population with respect to PGF and PT. Based on the reported +2m incidence of PGF and PT of 30% for non-prophylaxis post-transplantation in acute leukemia with BM EC< 0.1% pre-haplo-HSCT, a clinically meaningful reduction in the +2m incidence of PGF and PT would be 10% for NAC prophylaxis peri-transplantation.

11.2.1 Sample Size Assumptions

The sample size calculation is based on the primary endpoint, the +2m incidence of PGF and PT post-transplantation, with the following assumptions:

- the +2m incidence of PGF and PT in the control group: 30%
- the +2m incidence of PGF and PT in the NAC group: 10%
- a 2:1 randomization scheme
- a 5% one-tailed risk of erroneously claiming a difference in the presence of no true underlying difference by z-test with pooled variance
- a 80% chance of successfully declaring a difference in the presence of a true underlying difference (power)
- 10% percent of cases drop

Under the above assumptions, a total sample size of 130 subjects is required (87 in NAC group and 43 in control).

11.2.2 Primary Efficacy Endpoint

The primary efficacy endpoint is the +2m incidence of PGF and PT post-transplantation.

11.2.3 Secondary Efficacy Endpoints

The secondary efficacy endpoints include OS and LFS.

11.3 Data Analysis Considerations

11.3.1 Analysis Population

The primary population will be the intent-to-treat (ITT) population, which is defined as all subjects randomized to the two groups. This ITT population will be the basis for the analysis of efficacy and safety endpoints in this study.

11.3.2 Analysis Plan

11.3.2.1 Baseline Data

Baseline characteristics will be summarized and described in a frequency list.

11.3.2.2 Analysis of Efficacy

The definition of efficacy endpoints has been detailed in previous section. PGF was defined as the presence of 2 or 3 cytopenic counts (ANC≤0.5×109/L, platelet≤20×109/L, or hemoglobin≤70 g/L) for at least 3 consecutive days beyond day +28 post-HSCT with a transfusion requirement, related with hypoplastic-aplastic BM, in the presence of complete donor chimerism (CDC). PT was defined as platelet count less than 20×10^9/L or a dependence on platelet transfusion with the engraftment of other cell lines(ANC>0.5×10^9/L and hemoglobin>70 g/L without transfusion support) beyond day +60 post-HSCT in the presence of CDC.

Cumulative incidences of PGF/PT, myeloid and platelet engraftment, relapse, NRM, and GVHD were calculated by accounting for competing risks using the Fine and Gray model. Cumulative incidence curves were used in a competing risk setting, with relapse treated as a competing event, to calculate NRM probabilities, and with death from any cause as a competing risk for PGF/PT, engraftment, GVHD, EBV or CMV reactivation, and relapse. OS and LFS are estimated using the Kaplan-Meier method and compared using the log-rank test. The corresponding HR and 95% CI were estimated using the Cox proportional hazards model. All statistical tests are two-tailed with a significance level of 0.05. SPSS 20.0 (SPSS Inc., Chicago, IL, USA) and R version 3.3.0 (R Development Core Team, Vienna, Austria) are used for all data analysis.

11.3.2.3 Analysis of Safety

Safety and tolerability will be assessed by incidence and severity of AEs and changes from baseline of all relevant parameters, including laboratory test values,

physical examination, vital signs, and ECOG performance scores. The definition of AEs has been detailed in previous section. With the exception of hematologic AEs, all AEs are graded according to CTCAE version 4.0. All subjects will be monitored for AEs within 60 days post-transplantation. Categorical data will be summarized by proportion of total subjects. Quantitative data will be described using arithmetic average or median for central tendency and standard deviation or interquartile range for distribution range.

12. Materials for the Study

All materials provided to study sites and investigators are as follows:

- The study protocol
- Informed consent
- CRF

13. Ethical Considerations

13.1 Responsibility of Investigators

The investigators have the responsibility for guarantee of the clinical study's compliance with the protocol, Chinese good clinical practice (GCP) guidelines and applicable laws and regulations.

13.2 Informed Consent Process

Prior to participation in the study, subjects must be informed about objectives, methods, possible benefits, potential risks and possible discomforts of the study by investigators. They also should be informed that participation in the study would be voluntary, they can withdraw from the study at any time, there is no impact on the treatment of the disease whether they take part in the study and their privacy will be

protected.

Subjects or their legally acceptable representative should have enough time to read the inform consent and raise queries. Written informed consent must be obtained from each subject, or their legally acceptable representative.

13.3 Good Clinical Practice

This study will be conducted in accordance with the Declaration of Helsinki and Chinese GCP. The study will be conducted only if it is approved by the ethical review committee of the principal study site. The investigators will guarantee that the study will be conducted in accordance with applicable laws and regulations, scientific and ethical principles of the People's Republic of China. If the protocol needs revision during the study, the revised version must be reapproved by the ethical review committee of the principal study site. If new data related to study treatment are discovered, the informed consent must be revised and the revision must be reapproved by the ethical review committee of the principal study site and subjects.

13.4 Protection of Subjects' Personal Data

Data collected in the study are limited to the efficacy and safety related to study treatment. Data will be collected and used in accordance with applicable laws and regulations.

14. Administrative Requirements

Neither the investigator nor the applicant can revise the protocol without agreement of the opposite side. All revisions of the protocol must be released by the applicant institution. To insure the integrity, accuracy and reliability of the data, relevant results of examination and treatment must be documented in original medical record and CRF. Independent clinical monitoring is performed regularly by a panel of qualified and

experienced study investigators composed of hematologists who are blinded as to the treatment assignments.

15. References

1. Kong Y, Chang YJ, Wang YZ, et al. Association of an impaired bone marrow microenvironment with secondary poor graft function after allogeneic hematopoietic stem cell transplantation. *Biol Blood Marrow Transplant*. 2013;19(10):1465-1473.

2. Kong Y, Wang YT, Hu Y, et al. The bone marrow microenvironment is similarly impaired in allogeneic hematopoietic stem cell transplantation patients with early and late poor graft function. *Bone Marrow Transplant*. 2016;51(2):249-255.

3. Kong Y, Song Y, Hu Y, et al. Increased reactive oxygen species and exhaustion of quiescent CD34-positive bone marrow cells may contribute to poor graft function after allotransplants. *Oncotarget*. 2016;7(21):30892-30906.

4. Shi MM, Kong Y, Song Y, et al. Atorvastatin enhances endothelial cell function in posttransplant poor graft function. *Blood*. 2016;128(25):2988-2999.

5. Zhao HY, Lyu ZS, Duan CW, et al. An unbalanced monocyte macrophage polarization in the bone marrow microenvironment of patients with poor graft function after allogeneic haematopoietic stem cell transplantation. *Br J Haematol*. 2018;182(5):679-692.

6. Kong Y, Shi MM, Zhang YY, et al. N-acetyl-L-cysteine improves bone marrow endothelial progenitor cells in prolonged isolated thrombocytopenia patients post allogeneic hematopoietic stem cell transplantation. *Am J Hematol*. 2018;93(7):931-942.

7. Song Y, Shi MM, Zhang YY, et al. Abnormalities of the Bone Marrow Immune Microenvironment in Patients with Prolonged Isolated Thrombocytopenia after Allogeneic Hematopoietic Stem Cell Transplantation. *Biol Blood Marrow Transplant*. 2017;23(6):906-912.

8. Kong Y, Hu Y, Zhang XH, et al. Association between an impaired bone marrow vascular microenvironment and prolonged isolated thrombocytopenia after allogeneic hematopoietic stem cell transplantation. *Biol Blood Marrow Transplant*. 2014;20(8):1190-1197.

9. Olsson RF, Logan BR, Chaudhury S, et al. Primary graft failure after myeloablative allogeneic hematopoietic cell transplantation for hematologic malignancies. *Leukemia*. 2015;29(8):1754-1762.

10. Chang YJ, Zhao XY, Xu LP, et al. Donor-specific anti-human leukocyte antigen antibodies were associated with primary graft failure after unmanipulated haploidentical blood and marrow transplantation: a prospective study with randomly assigned training and validation sets. *J Hematol Oncol*. 2015;8:84.

11. Reisner Y, Hagin D, Martelli MF. Haploidentical hematopoietic transplantation: current

status and future perspectives. *Blood*. 2011;118(23):6006-6017.

12. Kong Y. Poor graft function after allogeneic hematopoietic stem cell transplantation—an old complication with new insights.

13. Scadden DT. The stem-cell niche as an entity of action. *Nature*. 2006;441(7097):1075-1079.

14. Calvi LM, Adams GB, Weibrecht KW, et al. Osteoblastic cells regulate the haematopoietic stem cell niche. *Nature*. 2003;425(6960):841-846.

15. Ding L, Saunders TL, Enikolopov G, Morrison SJ. Endothelial and perivascular cells maintain haematopoietic stem cells. *Nature*. 2012;481(7382):457-462.

16. Morrison SJ, Scadden DT. The bone marrow niche for haematopoietic stem cells. *Nature*. 2014;505(7483):327-334.

17. Crane GM, Jeffery E, Morrison SJ. Adult haematopoietic stem cell niches. *Nat Rev Immunol*. 2017;17(9):573-590.

18. Hooper AT, Butler JM, Nolan DJ, et al. Engraftment and reconstitution of hematopoiesis is dependent on VEGFR2-mediated regeneration of sinusoidal endothelial cells. *Cell Stem Cell*. 2009;4(3):263-274.

19. Bautch VL. Stem cells and the vasculature. *Nat Med*. 2011;17(11):1437-1443.

20. Bao XM, Wu CF, Lu GP. Atorvastatin inhibits homocysteine-induced oxidative stress and apoptosis in endothelial progenitor cells involving Nox4 and p38MAPK. *Atherosclerosis*. 2010;210(1):114-121.

21. Cai H, Harrison DG. Endothelial dysfunction in cardiovascular diseases: the role of oxidant stress. *Circ Res*. 2000;87(10):840-844.

22. Motohashi H, Kimura M, Fujita R, et al. NF-E2 domination over Nrf2 promotes ROS accumulation and megakaryocytic maturation. *Blood*. 2010;115(3):677-686.

23. Ito K, Hirao A, Arai F, et al. Reactive oxygen species act through p38 MAPK to limit the lifespan of hematopoietic stem cells. *Nat Med*. 2006;12(4):446-451.

24. Hu L, Cheng H, Gao Y, et al. Antioxidant N-acetyl-L-cysteine increases engraftment of human hematopoietic stem cells in immune-deficient mice. *Blood*. 2014;124(20):e45-48.

25. Dodd S, Dean O, Copolov DL, Malhi GS, Berk M. N-acetylcysteine for antioxidant therapy: pharmacology and clinical utility. *Expert Opin Biol Ther*. 2008;8(12):1955-1962.

26 Kong Y, Wang Y, Zhang YY, et al. Prophylactic oral NAC reduced poor hematopoietic reconstitution by improving endothelial cells after haploidentical transplantation. Blood Adv. 2019 Apr 23;3(8):1303-1317

27. Werner N, Kosiol S, Schiegl T, et al. Circulating endothelial progenitor cells and cardiovascular outcomes. *N Engl J Med*. 2005;353(10):999-1007.

28. Duan CW, Shi J, Chen J, et al. Leukemia propagating cells rebuild an evolving niche

in response to therapy. *Cancer Cell*. 2014;25(6):778-793.

29. Nachman RL, Rafii S. Platelets, petechiae, and preservation of the vascular wall. *N Engl J Med*. 2008;359(12):1261-1270.

30. Lin Y, Weisdorf DJ, Solovey A, Hebbel RP. Origins of circulating endothelial cells and endothelial outgrowth from blood. *J Clin Invest*. 2000;105(1):71-77.

31. Simmons PJ, Przepiorka D, Thomas ED, Torok-Storb B. Host origin of marrow stromal cells following allogeneic bone marrow transplantation. *Nature*. 1987;328(6129):429-432.

32. Salter AB, Meadows SK, Muramoto GG, et al. Endothelial progenitor cell infusion induces hematopoietic stem cell reconstitution in vivo. *Blood*. 2009;113(9):2104-2107.

16 Appendices

16.1 Appendix 1 Diagnosis and Classification of aGVHD and cGVHD

Grading of aGVHD

Grade	Degree of organ involvement
I	Stage 1-2 skin rash; no gut involvement; no liver involvement; no decrease in clinical performance
II	Stage 1-3 skin rash; stage 1 gut involvement or stage 1 liver involvement (or both); mild decrease in clinical performance
III	Stage 2-3 skin rash; stage 2-3 gut involvement or 2-4 liver involvement (or both); marked decrease in clinical performance
IV	Similar to Grade III with stage 2-4 organ involvement and extreme decrease in clinical performance

Przepiorka D, Weisdorf D, Martin P, Klingemann HG, Beatty P, Hows J, Thomas ED. 1994 Consensus Conference on Acute GVHD Grading. Bone Marrow Transplant. 1995; 15(6):825-828.

Grading of cGVHD

NIH Global Severity of chronic GVHD

Mild chronic GVHD

1 or 2 Organs involved with no more than score 1 plus Lung score 0

Moderate chronic GVHD

3 or More organs involved with no more than score 1

OR

At least 1 organ (not lung) with a score of 2

OR

Lung score 1

Severe chronic GVHD

At least 1 organ with a score of 3

OR

Lung score of 2 or 3

Key points:

In skin: higher of the 2 scores to be used for calculating global severity.

In lung: FEV1 is used instead of clinical score for calculating global severity.

If the entire abnormality in an organ is noted to be unequivocally explained by a non-GVHD documented cause, that organ is not included for calculation of the global severity.

If the abnormality in an organ is attributed to multifactorial causes (GVHD plus other causes), the scored organ will be used for calculation of the global severity regardless of the contributing causes (no downgrading of organ severity score).

Jagasia MH, Greinix HT, Arora M, Williams KM, Wolff D, Cowen EW, Palmer J, Weisdorf D, Treister NS, Cheng GS, Kerr H, Stratton P, Duarte RF, McDonald GB, Inamoto Y, Vigorito A, Arai S, Datiles MB, Jacobsohn D, Heller T, Kitko CL, Mitchell SA, Martin PJ, Shulman H, Wu RS, Cutler CS, Vogelsang GB, Lee SJ, Pavletic SZ, Flowers ME. National Institutes of Health Consensus Development Project on Criteria for Clinical Trials in Chronic Graft-versus-Host Disease: I. The 2014 Diagnosis and Staging Working Group report. Biol Blood Marrow Transplant. 2015; 21(3): 389-401.e1.

	SCORE 0	SCORE 1	SCORE 2	SCORE 3
**PERFORMANCE SCORE:** ☐ **KPS ECOG LPS**	☐ Asymptomatic and fully active (ECOG 0; KPS or LPS 100%)	☐ Symptomatic, fully ambulatory, restricted only in physically strenuous activity (ECOG 1, KPS or LPS 80-90%)	☐ Symptomatic, ambulatory, capabl of self-care, >50% of waking hours ou of bed (ECOG 2, KPS or LPS 60-70%)	☐ Symptomatic, limited self-care, >50% of waking hours in bed (ECOG 3-4, KPS or LPS <60%)
**SKIN†** **SCORE % BSA** ☐ *GVHD features to be scored by BSA:* **Check all that apply:** ☐ Maculopapular rash/erythema ☐ Lichen planus-like features ☐ Sclerotic features ☐ Papulosquamous lesions or ichthyosis ☐ Keratosis pilaris-like GVHD	☐ No BSA involved	☐ 1-18% BSA	☐ 19-50% BSA	☐ >50% BSA
**SKIN FEATURES SCORE:**	☐ No sclerotic features		☐ Superficial sclerotic features "not hidebound" (able to pinch)	**Check all that apply:** ☐ Deep sclerotic features ☐ "Hidebound" (unable to pinch) ☐ Impaired mobility ☐ Ulceration
Other skin GVHD features (NOT scored by BSA) **Check all that apply:** ☐ Hyperpigmentation ☐ Hypopigmentation ☐ Poikiloderma ☐ Severe or generalized pruritus ☐ Hair involvement ☐ Nail involvement ☐ *Abnormality present but explained entirely by non-GVHD documented cause (specify):*______________________________				
**MOUTH** *Lichen planus-like features present:* ☐ **Yes** ☐ **No**	☐ No symptoms	☐ Mild symptoms **with** disease signs but not limiting oral intake significantly	☐ Moderate symptoms with disease signs **with** partial limitation of oral intake	☐ Severe symptoms with disease signs on examination **with** major limitation of oral intake
☐ *Abnormality present but explained entirely by non-GVHD documented cause (specify):*______________________________				

Figure 1. Organ scoring of chronic GVHD. ECOG indicates Eastern Cooperative Oncology Group; KPS, Karnofsky Performance Status; LPS, Lansky Performance Status; BSA, body surface area; ADL, activities of daily living; LFTs, liver function tests; AP, alkaline phosphatase; ALT, alanine aminotransferase; ULN, normal upper limit. *Weight loss within 3 months. †Skin scoring should use both percentage of BSA involved by disease signs and the cutaneous features scales. When a discrepancy exists between the percentage of total body surface (BSA) score and the skin feature score, OR if superficial sclerotic features are present (Score 2), but there is impaired mobility or ulceration (Score 3), the higher level should be used for the final skin scoring. **Lung scoring should be performed using both the symptoms and FEV1 scores whenever possible. FEV1 should be used in the final lung scoring where there is discrepancy between symptoms and FEV1 scores.

	SCORE 0	SCORE 1	SCORE 2	SCORE 3
EYES<br><br>*Keratoconjunctivitis sicca (KCS) confirmed by ophthalmologist:*<br>☐ **Yes**<br>☐ **No**<br>☐ **Not examined**	☐ No symptoms	☐ Mild dry eye symptoms not affecting ADL (requirement of lubricant eye drops ≤ 3 x per day)	☐ Moderate dry eye symptoms partially affecting ADL (requiring lubricant eye drops > 3 x per day or punctal plugs), **WITHOUT** new vision impairment due to KCS	☐ Severe dry eye symptoms significantly affecting ADL (special eyeware to relieve pain) **OR** unable to work because of ocular symptoms **OR** loss of vision due to KCS
☐ *Abnormality present but explained entirely by non-GVHD documented cause (specify):*______________________________				
**GI Tract**<br>***Check all that apply:***<br>☐ Esophageal web/ proximal stricture or ring<br>☐ Dysphagia<br>☐ Anorexia<br>☐ Nausea<br>☐ Vomiting<br>☐ Diarrhea<br>☐ Weight loss ≥5%*<br>☐ Failure to thrive	☐ No symptoms	☐ Symptoms without significant weight loss* (<5%)	☐ Symptoms associated with mild to moderate weight loss* (5-15%) **OR** moderate diarrhea without significant interference with daily living	☐ Symptoms associated with significant weight loss* >15%, requires nutritional supplement for most calorie needs **OR** esophageal dilation **OR** severe diarrhea with significant interference with daily living
☐ *Abnormality present but explained entirely by non-GVHD documented cause (specify):*______________________________				
LIVER	☐ Normal total bilirubin and ALT or AP < 3 x ULN	☐ Normal total bilirubin with ALT ≥3 to 5 x ULN or AP ≥ 3 x ULN	☐ Elevated total bilirubin but ≤3 mg/dL or ALT > 5 ULN	☐ Elevated total bilirubin > 3 mg/dL
☐ *Abnormality present but explained entirely by non-GVHD documented cause (specify):*				
LUNGS**<br>**Symptom score**:	☐ No symptoms	☐ Mild symptoms (shortness of breath after climbing one flight of steps)	☐ Moderate symptoms (shortness of breath after walking on flat ground)	☐ Severe symptoms (shortness of breath at rest; requiring 0_2)
**Lung score**:<br>**%** FEV1 ☐<br><br>*Pulmonary function tests*<br>☐ Not performed	☐ FEV1≥80%	☐ FEV1 60-79%	☐ FEV1 40-59%	☐ FEV1 ≤39%
☐ *Abnormality present but explained entirely by non-GVHD documented cause (specify):*______________________________				

Figure 1. (continued).

	SCORE 0	SCORE 1	SCORE 2	SCORE 3
**JOINTS AND FASCIA**<br><br>P-ROM score<br>*(see below)*<br>Shoulder (1-7):___<br>Elbow (1-7): ____<br>Wrist/finger (1-7):___<br>Ankle (1-4):___	☐ No symptoms	☐ Mild tightness of arms or legs, normal or mild decreased range of motion (ROM) **AND** not affecting ADL	☐ Tightness of arms or legs **OR** joint contractures, erythema thought due to fasciitis, moderate decrease ROM **AND** mild to moderate limitation of ADL	☐ Contractures **WITH** significant decrease of ROM ***AND*** significant limitation of ADL (unable to tie shoes, button shirts, dress self etc.)
☐ *Abnormality present but explained entirely by non-GVHD documented cause (specify):*______________________				
**GENITAL TRACT**<br>(*See Supplemental figure*[‡])<br>☐ Not examined<br>*Currently sexually active*<br>☐ Yes<br>☐ No	☐ No signs	☐ Mild signs[‡] and females with or without discomfort on exam	☐ Moderate signs[‡] and may have symptoms with discomfort on exam	☐ Severe signs[‡] with or without symptoms
☐ *Abnormality present but explained entirely by non-GVHD documented cause (specify):*				

Other indicators, clinical features or complications related to chronic GVHD (check all that apply and assign a score to severity (0-3) based on functional impact where applicable none – 0,mild -1, moderate -2, severe – 3)

☐ Ascites (serositis)___	☐ Myasthenia Gravis___	
☐ Pericardial Effusion___	☐ Peripheral Neuropathy___	☐ Eosinophilia > 500/µl___
☐ Pleural Effusion(s)___	☐ Polymyositis___	☐ Platelets <100,000/µl ___
☐ Nephrotic syndrome___	☐ Weight loss>5%* without GI symptoms ___	☐ Others (specify):____________

**Overall GVHD Severity**<br>*(Opinion of the evaluator)*	☐ **No GVHD**	☐ **Mild**	☐ **Moderate**	☐ **Severe**

Photographic Range of Motion (P-ROM)

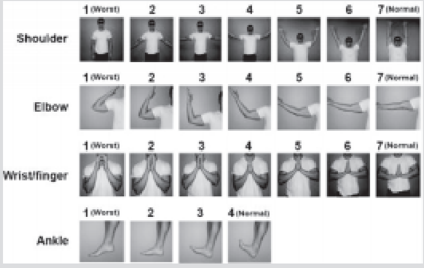

Figure 1. (continued).

Jagasia MH, Greinix HT, Arora M, Williams KM, Wolff D, Cowen EW, Palmer J, Weisdorf D, Treister NS, Cheng GS, Kerr H, Stratton P, Duarte RF, McDonald GB, Inamoto Y, Vigorito A, Arai S, Datiles MB, Jacobsohn D, Heller T, Kitko CL, Mitchell SA, Martin PJ, Shulman H, Wu RS, Cutler CS, Vogelsang GB, Lee SJ, Pavletic SZ, Flowers ME. National Institutes of Health Consensus Development Project on Criteria for Clinical Trials in Chronic Graft-versus-Host Disease: I. The 2014 Diagnosis and Staging Working Group report. Biol Blood Marrow Transplant. 2015; 21(3): 389-401.e1.

16.2 Appendix 2

ECOG Performance Status

**Grade**	**ECOG**
**0**	Fully active, able to carry on all pre-disease performance without restriction
**1**	Restricted in physically strenuous activity but ambulatory and able to carry out work of a light or sedentary nature, e.g., light house work, office work
**2**	Ambulatory and capable of all selfcare but unable to carry out any work activities. Up and about more than 50% of waking hours
**3**	Capable of only limited selfcare, confined to bed or chair more than 50% of waking hours
**4**	Completely disabled. Cannot carry on any selfcare. Totally confined to bed or chair
**5**	Dead

Oken MM, Creech RH, Tormey DC, Horton J, Davis TE, McFadden ET, Carbone PP. Toxicity And Response Criteria Of The Eastern Cooperative Oncology Group. Am J Clin Oncol. 1982; 5(6):649-655.
